# Subtle Molecular Changes Largely Modulate Chiral Helical Assemblies of Achiral Conjugated Polymers by Tuning Solution-State Aggregation

**DOI:** 10.1021/acscentsci.3c00775

**Published:** 2023-11-13

**Authors:** Kyung Sun Park, Xuyi Luo, Justin J. Kwok, Azzaya Khasbaatar, Jianguo Mei, Ying Diao

**Affiliations:** † Department of Chemical and Biomolecular Engineering, University of Illinois at Urbana−Champaign, 600 S. Mathews Ave., Urbana, Illinois 61801, United States; ‡ Department of Chemistry, Purdue University, 560 Oval Dr., West Lafayette, Indiana 47907, United States; § Department of Materials Science and Engineering, University of Illinois at Urbana−Champaign, 1304 W. Green St., Urbana, Illinois 61801, United States; ∥ Beckman Institute, Molecular Science and Engineering, University of Illinois at Urbana−Champaign, 405 N. Mathews Ave., Urbana, Illinois 61801, United States; ⊥ Department of Chemistry, University of Illinois at Urbana−Champaign, 505 S. Mathews Ave., Urbana, Illinois 61801, United States; # Materials Research Laboratory, The Grainger College of Engineering, University of Illinois at Urbana−Champaign, 104 S. Goodwin Ave., Urbana, Illinois 61801, United States

## Abstract

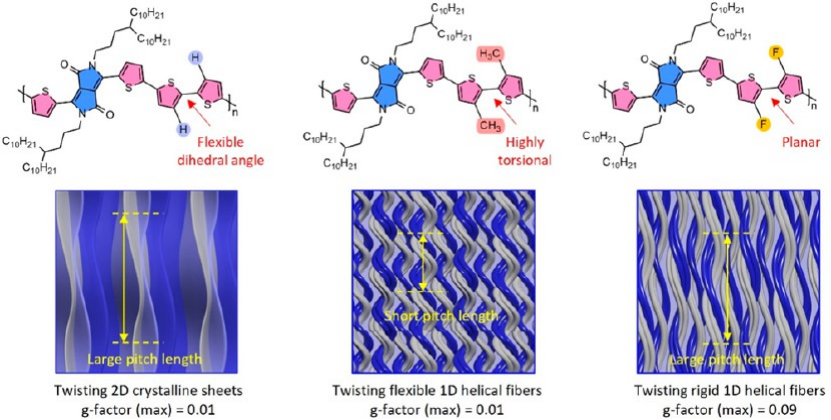

Understanding the solution-state aggregate structure and the consequent hierarchical assembly of conjugated polymers is crucial for controlling multiscale morphologies during solid thin-film deposition and the resultant electronic properties. However, it remains challenging to comprehend detailed solution aggregate structures of conjugated polymers, let alone their chiral assembly due to the complex aggregation behavior. Herein, we present solution-state aggregate structures and their impact on hierarchical chiral helical assembly using an achiral diketopyrrolopyrrole-quaterthiophene (DPP-T4) copolymer and its two close structural analogues wherein the bithiophene is functionalized with methyl groups (DPP-T2M2) or fluorine atoms (DPP-T2F2). Combining in-depth small-angle X-ray scattering analysis with various microscopic solution imaging techniques, we find distinct aggregate in each DPP solution: (i) semicrystalline 1D fiber aggregates of DPP-T2F2 with a strongly bound internal structure, (ii) semicrystalline 1D fiber aggregates of DPP-T2M2 with a weakly bound internal structure, and (iii) highly crystalline 2D sheet aggregates of DPP-T4. These nanoscopic aggregates develop into lyotropic chiral helical liquid crystal (LC) mesophases at high solution concentrations. Intriguingly, the dimensionality of solution aggregates largely modulates hierarchical chiral helical pitches across nanoscopic to micrometer scales, with the more rigid 2D sheet aggregate of DPP-T4 creating much larger pitch length than the more flexible 1D fiber aggregates. Combining relatively small helical pitch with long-range order, the striped twist-bent mesophase of DPP-T2F2 composed of highly ordered, more rigid 1D fiber aggregate exhibits an anisotropic dissymmetry factor (g-factor) as high as 0.09. This study can be a prominent addition to our knowledge on a solution-state hierarchical assembly of conjugated polymers and, in particular, chiral helical assembly of achiral organic semiconductors that can catalyze an emerging field of chiral (opto)electronics.

## Abstract 2

Small changes can have a big impact: Subtle changes in molecular structure of conjugated polymers sensitively tune chiral helical assembly through modulating solution-state aggregation.

## Introduction

Conjugated polymers have attracted intense interest for their numerous applications such as electronics,^1^ solar cells,^2^ thermoelectrics,^3^ photocatalysts,^4^ electrochemical devices^5^ and biomedical devices.^6^ One of the key advantages of conjugated polymers is their economic/energy efficient solution-processability, which directs the community to develop many innovative ideas on solution-processing techniques and compatible molecular designs.^7−9^ Ultimate optical and electronic properties of solid-state conjugated polymers are direct consequences of their multiscale morphology developed through complex and hierarchical assembly.^7^ Therefore, an in-depth exploration of polymer solution aggregation and mesophase assembly is critically important to understand and control the structure of conjugated polymers at all length scales. Semicrystalline fibril-like or network solution aggregates are commonly reported for most donor–acceptor (D–A) conjugated copolymers, facilitated through backbone and/or lamellar stacking via π–π and van der Waals interactions, respectively.^10^ The shape, size, and extent to which the polymer aggregation occurs in solution are affected by various factors, e.g., backbone planarity,^11^ regioregularity,^12^ and side-chain chemistry^13^ in molecular designs and solvent,^14^ temperature,^15^ and aging time^16^ in processing conditions. Recent advances have showcased how solution-state aggregates affect the degree of chain alignment, crystallinity and molecular orientation in solid-state thin films of direct relevance to device performance.^10^ In particular, simply tuning solvent quality/selectivity has afforded diverse solution-state aggregates with distinct internal structures.^14,17^

Recently, our group has discovered that semicrystalline fibrillar aggregates of an achiral isoindigo-based D–A conjugated polymer can assemble into lyotropic chiral helical liquid crystalline (LC) phases upon increasing solution concentration.^18^ Briefly, the twist and bent intramolecular conformation and staggered intermolecular stacking enable the formation of chiral helical nanofibers, which, in turn, give rise to chiral LC mesophases. Furthermore, this chiral LC mesophase emergence is sensitive to solvent selectivity^14^—chiral mesophases only form in solvents (e.g., chlorobenzene, dichlorobenzene, toluene) mutually dissolving the polymer backbone and the side chain but not in solvents selectively dissolving either the backbone (chloronaphthalene) or the side chain (decane). We further showed that varying the solvent selectivity alters the solution aggregate structures, thereby controlling the assembly pathways. Specifically, a backbone selective solvent leads to side-chain associated amorphous network aggregates, which give rise to a direct crystallization pathway during solution printing to yield highly aligned thin films. On the other hand, side chain selective solvent engenders semicrystalline fibers featuring strong but disordered π–π stacking; strong interfiber interaction leads to massive agglomeration assembly pathway resulting in almost isotropic thin films. Interestingly, only mutual solvents lead to helical fiber aggregates, which deliver a chiral mesophase mediated assembly pathway and resultant chiral twinned morphology in printed films.

Chiral supramolecular structures,^19^ developed from the hierarchical assembly of chiral molecular components and/or achiral building blocks, have attracted intense interests across supramolecular chemistry and materials science because of their alluring potential applications, such as the production or detection of circularly polarized light,^20−22^ chiral electrochemical sensors,^23^ chiroptical switching in information storage,^20^ and biomolecular sensing.^24^ General approaches to developing chiral structures of conjugated polymers are assembly of chiral conjugated polymers by introducing chiral units on polymer backbones^28^ or side chains^29^ and assembly of achiral conjugated polymers with chiral additives.^29−31^ Chiral assemblies from common achiral building blocks are rare but compelling, as it opens up the vast chemical design space. However, it remains unclear what molecular attributes determine the chiral emergence and assembly pathway of achiral building blocks.

In this work, we vary the molecular structure of diketopyrrolopyrrole-based copolymers in subtle ways and discover that the molecular structure sensitively tunes chiral helical assemblies through modulation of the structure of solution-state aggregates. We first investigate the solution-state aggregate structures of a diketopyrrolopyrrole-quaterthiophene copolymer (DPP-T4) and its two close derivatives, DPP-T2M2 and DPP-T2F2. We report that DPP-T2M2 and DPP-T2F2 solutions constitute semicrystalline 1D fiber aggregates mixed with amorphous networks of single polymer chains, whereas DPP-T4 solutions are composed of 2D sheet crystalline aggregates. Moreover, we observe lyotropic chiral LC mesophases, in which each DPP aggregate is developed into multiscale helical structures with distinct nano/micrometer helical pitch length scales tuned by the dimensionality of solution aggregates. Such complex hierarchical assembly is unveiled by combining X-ray scattering, optical, electron, and atomic force microscopy imaging, and optical spectroscopy, together with DFT calculations.

## Results and Discussion

**Figure 1:**
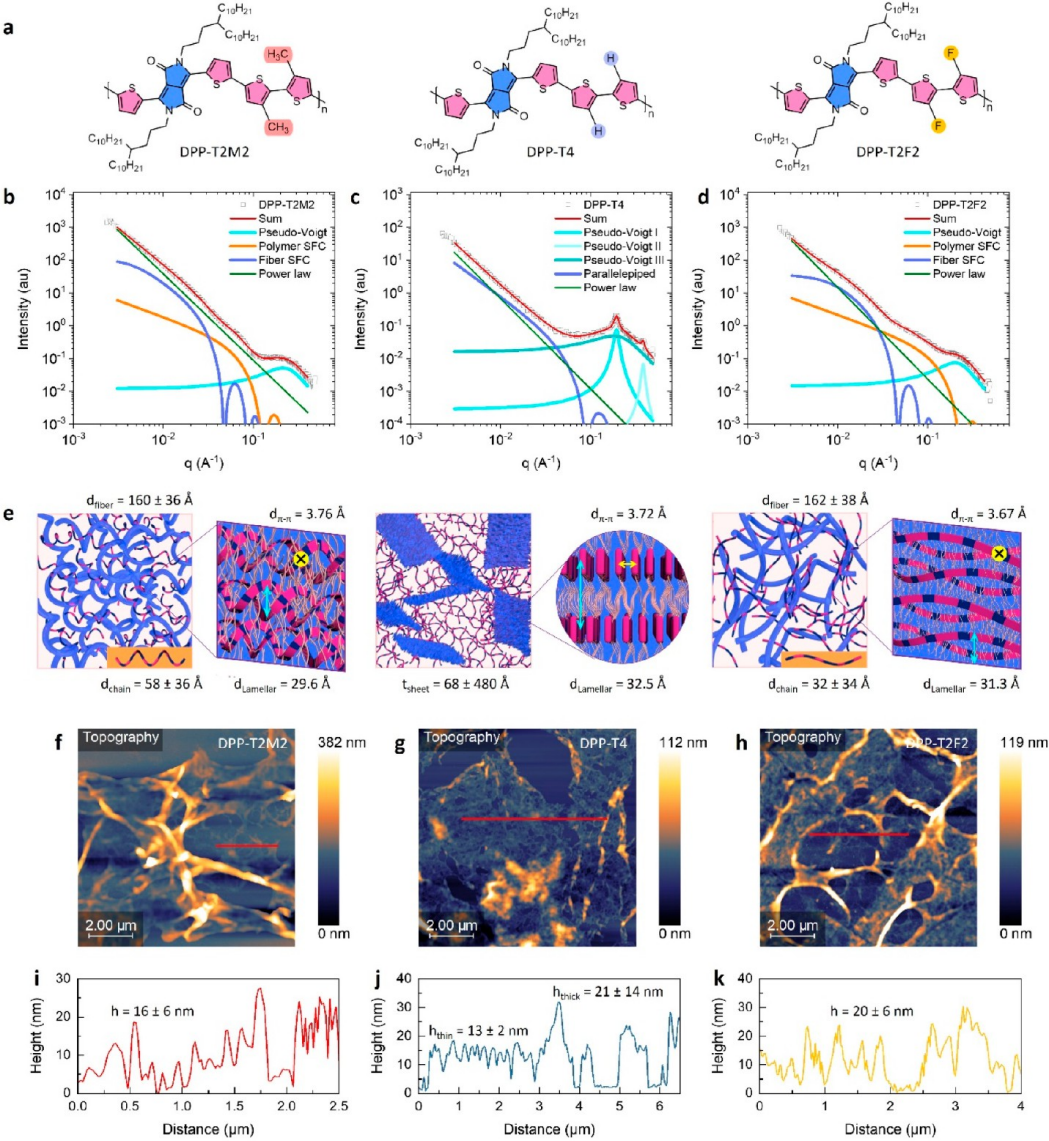
Distinct solution aggregation of DPP-based copolymers. (a) Molecular structures of DPP-T2M2, DPP-T4, and DPP-T2F2. The difference of the functional component is highlighted. (b, d) SAXS 1D profiles of DPP polymer solutions made in CB at 30 mg/mL. Colored solid lines are fitting results using the 2SFC model for DPP-T2M2 and DPP-T2F2, and the SA model is for DPP-T4. (e) Schematic illustration of the solution structure in each DPP system (left) and presumed internal structures of aggregation (right). The blue (aggregates), orange (chains), and cyan (lamellar stacking) color codes in the scheme match with each corresponding fitting line of the SAXS panels. The yellow dot or arrow indicates the π–π stacking which distance is obtained in the WAXS region (Figure S1). (f–h) AFM images of freeze-dried samples prepared from the solution at the same concentration taken by SAXS. (i–k) AFM height profiles of the corresponding region drawn by red lines on the AFM images.

Figure 1a shows molecular structures of DPP systems that are designed to tune backbone torsion with methyl or fluorine substitution on the bithiophene unit. The polymers were synthesized via Stille coupling from a DPP-bithiophene monomer with each corresponding bithiophene ditin compound (see Materials and Methods).^32,33^ In our previous study,^18^ it was found that the highly flexible thiophene-thiophene (T-T) dihedral in the isoindigo-bithiophene-based conjugated polymer is critical to forming wavy, helical polymer chain conformation. Such polymer chains further stack in a staggered fashion to form chiral helical fibrils in solution. Here, we investigate how varying the torsional angle of T-T units in DPP systems can affect the solution state aggregation and thus the chiral helical assembly. Detailed molecular conformations were characterized in depth using DFT calculations and optical and vibrational spectroscopy, discussed later.

First, we investigate the solution-state structures of three DPP systems by characterizing the polymer conformation and intraaggregate packing using small-angle X-ray scattering (SAXS). SAXS samples were prepared by dissolving polymers in chlorobenzene (30 mg/mL) and injecting the solutions into glass capillaries. Figure 1b–1d shows 1D SAXS scattering profiles with fitting analysis of DPP solution systems. All solutions show a broad peak in the high q-region around 0.19–0.21 Å^–1^ which corresponds to lamellar stacking within the polymer aggregates.^34^ Notably, DPP-T4 shows additional sharp first- and second-order scattering peaks at 0.19 and 0.38 Å^–1^, respectively (Figure 1c), indicating the highly ordered lamellar structures in the solution aggregates. Also, in all samples, a power law slope of around −2.7 was observed at the low q-region, suggesting Porod scattering from a larger network-like feature composed of polymer chains, aggregates, or both. In the case of DPP-T2M2 and DPP-T2F2, the SAXS profiles are quite similar, consisting of two Guinier knee regions at around 0.02 and 0.08 Å^–1^, with the broad lamellar peak. In fact, this particular SAXS scattering feature has been commonly observed when D–A conjugated polymers form fibrillar aggregates in a solution state.^14,35^ The recent SAXS fitting model we developed for such fibril aggregation system (called as the 2SFC model)^35^ where the aggregates coexist with dispersed polymer chains in solution was thus suited for analyzing DPP-T2M2 and DPP-T2F2 systems. Detailed fitting parameters are summarized in Table S1. The DPP-T2M2 solution scattering profile is deconvoluted into four components (Figure 1b): (i) a pseudo-Voigt peak centered around 0.21 Å^–1^ indicating the presence of lamellar stacking with a distance of 29.9 Å, (ii) a semiflexible cylinder (SFC) fitted around 0.08 Å^–1^ representing dispersed polymer chains with a diameter of 58 ± 36 Å, (iii) another SFC fitted around 0.02 Å^–1^ representing fiber-like aggregates with a diameter of 160 ± 36 Å, and (iv) a power law slope of −2.6. Similarly, the DPP-T2F2 solution scattering profile is fitted to the same model (Figure 1d), resulting in a lamellar stacking distance of 31.3 Å, polymer chains with a diameter of 32 ± 34 Å, fibrillar aggregates with a diameter of 162 ± 38 Å, and a power law of −2.8. Moreover, the existence of π–π stacking in both DPP-T2M2 (d_π–π_ = 3.76 Å) and DPP-T2F2 (d_π–π_ = 3.67 Å) solution systems is confirmed in the wide-angle X-ray scattering (WAXS) region (Figure S1). This result points out that the solution aggregation of DPP-T2M2 and DPP-T2F2 take the form of semicrystalline fibrils with comparable diameters in the range of 160–200 Å. However, a difference between the DPP-T2M2 and DPP-T2F2 systems is clearly displayed in terms of the molecular conformation and internal structure of the aggregates. The cross section of DPP-T2F2 polymer chain (32 ± 34 Å) closely matches with the lamellar distance (31.3 Å) in the semicrystalline fibers, indicating almost fully extended polymer chains in tightly packed aggregates. On the other hand, the diameter of DPP-T2M2 polymer chains (58 ± 36 Å) is markedly larger than the lamellar distance of DPP-T2M2 fibers (29.6 Å). We attribute this larger DPP-T2M2 chain diameter to the increased effective volume of cylindrically shaped polymer chains as the molecular conformation twists more. This large difference in the internal structure of DPP-T2M2 and DPP-T2F2 solution aggregates is further proved by temperature-variant small and wide-angle X-ray scattering solution measurements (Figure S1); both lamellar and π–π stacking peaks are unchanged for the DPP-T2F2 solution during thermal annealing, whereas the decreased lamellar peak intensity and larger π–π stacking distance are observed for DPP-T2M2 upon heating.

Interestingly, the solution aggregation behavior for DPP-T4 is more distinct from those of DPP-T2M2 and DPP-T2F2 as seen in its sharp rather than broad lamellar stacking peaks as well as nearly absence of Guinier knee corresponding to dispersed polymer chains (Figure 1c). To explain this, we propose that DPP-T4 aggregation is a two-dimensional crystalline sheet resulting from strong interactions along both lamellar and π–π stacking directions. The WAXS scattering of DPP-T4 also confirms the presence of π-stacking in solution aggregate (Figure S1). Despite being almost featureless in the intermediate q-region for DPP-T4 in contrast to DPP-T2M2 and DPP-T2F2, the weak Guinier knee is found around 0.01 Å^–1^ when comparing the scattering of the solution annealed at 110 °C (Figure S1). This feature is attributed to the thickness of the crystalline sheet, as described below. The SAXS fitting model for DPP-T4 is slightly modified by including three pseudo-Voigt functions for fitting two sharp and one broad structure factor peaks, a parallelepiped model for fitting the form factor of 2D sheet-like aggregates, and a power law for fitting large-scale aggregates. We refer to it as the SA (sheet aggregation) model. Detailed fitting parameters are summarized in Table S2. The DPP-T4 solution scattering profile fits well with this model; two exceptionally high-ordered peaks at 0.19 and 0.38 Å^–1^ corresponding to the (100) and (200) lamellar stacking with a *d*-spacing of 32.5 and 16.4 Å, respectively, a broad peak centered around 0.18 Å^–1^ corresponding to another lamellar stacking distance of 34.7 Å, sheet-like aggregates with a thickness of 68 ± 480 Å, and a power law of −2.7. The error of the thickness is quite high due to the low scattering intensity; however, the presence of such aggregates is confirmed by imaging shown later. The two distinct populations of lamellar stackings indicate that two types of aggregates exist in DPP-T4 solution. Upon heating the DPP-T4 solution during X-ray scattering measurements, the sharp lamellar peaks and Guinier knee vanished; at the same time, the original broad lamella peak is left unchanged while the power law decay exponent changed from −2.7 to −3.4 (Figure S1). This suggests that upon heating, the crystalline 2D sheets dissolve to leave behind a network-like aggregate formed by single polymer chains. Both types of aggregates are present in the pristine solution before annealing. This in-depth X-ray scattering analysis allows us to depict a detailed solution structure of each DPP system (Figure 1e). DPP-T2M2 and DPP-T2F2 solutions are composed of semicrystalline 1D fiber aggregates coexisting with a network aggregate of dispersed polymer chains, whereas DPP-T4 consists of crystalline 2D sheet aggregates coexisting with a network aggregate of dispersed polymer chains. Furthermore, their internal structure exhibits notable diversity—we propose that DPP-T2M2 and DPP-T2F2 polymer chains are formed in a helical fashion within their fibrillar aggregates based on comprehensive structural characterizations discussed later.

Further direct imaging analysis supports the picture of solution aggregation portrayed above. AFM (Figure 1f–1h), SEM (Figure S2a–S2c), and TEM (Figure S2d–S2f) images were obtained from samples that were freeze-dried solutions at the same concentration of 30 mg/mL used for SAXS measurements. In the cases of DPP-T2M2 and DPP-T2F2, all images show apparent fibrillar aggregates. The fibrils of DPP-T2M2 aggregates shown in the TEM image (Figure S2d) seem more collapsed when compared to the relatively extended fibrils of DPP-T2F2 aggregates (Figure S2f). This observation is in line with the SAXS observation that loosely bonded DPP-T2M2 fibers may be more flexible, whereas the closely packed DPP-T2F2 fibers are likely rather rigid. The fibrillar width (*w*) and thickness (*t*) of DPP-T2M2 and DPP-T2F2 were obtained by measuring individually dispersed fibrils from each TEM and AFM images: *w=* 17 ± 4 nm and *t=* 16 ± 6 nm for DPP-T2M2 and *w* = 21 ± 3 nm and *t=* 20 ± 6 nm for DPP-T2F2. This indicates that the cross-section of the DPP-T2M2 and DPP-T2F2 fibers is nearly circular. Also, the measured values are in good agreement with the diameter of the aggregated fibers extracted from our SAXS measurements. Note that only individual fibrils were counted to estimate the thickness; large, agglomerated fibrils seen in the images are out of range for SAXS and contribute to the power law only at low *q*. Nonetheless, these large aggregates likely coexist in the solutions, as seen in all images. Noticeably, the SEM and TEM image of DPP-T4 aggregates appear as diffusive “clouds” rather than distinct fibrils in contrast to DPP-T2M2 and DPP-T2F2 (Figure S2). The AFM image of DPP-T4 (Figure 1g) additionally confirms the sheet-like aggregates, exhibiting thin and thick parts with a thickness of 13 ± 2 and 21 ± 14 nm, respectively. This value corresponds to the thickness range of DPP-T4 sheet-like aggregates extracted from the SAXS measurements.

**Figure 2:**
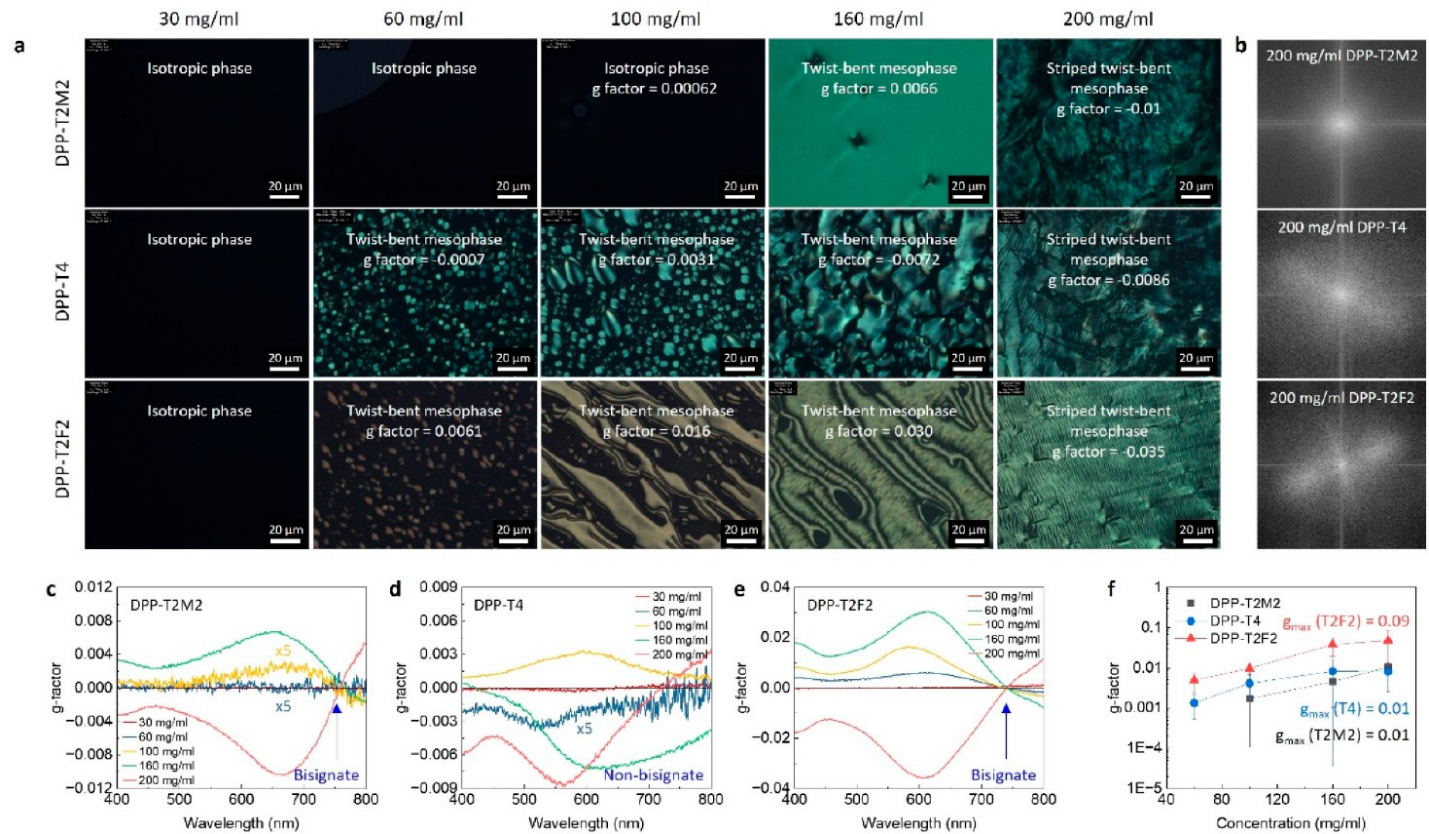
Chirality evolution of lyotropic DPP LC mesophase. (a) CPOM images of DPP-T2M2 (top), DPP-T4 (middle), and DPP-T2F2 (bottom) solutions using transmitted light, showing various morphologies of the LC mesophases. Each proposed phase and corresponding *g*-factor are denoted in the images. (b) FFT images of 200 mg/mL DPP mesophases shown in a. Apparent anisotropic character for DPP-T2F2 over the large area indicates the long-range ordered striped twist-bent phase when compared to DPP-T2M2 and DPP-T4. (c–e) CD spectra of the corresponding DPP solutions shown in a, indicating chirality emerges when exceeding a critical concentration at ≥100 mg/mL for DPP-T2M2, and ≥60 mg/mL for DPP-T4 and DPP-T2F2. (f) *g*-factor comparison as a function of solution concentration where the values are obtained from the maximum peak around 670, 580, and 600 nm for DPP-T2M2, DPP-T4, and DPP-T2F2, respectively. It is noted that the maximum g-factor value of DPP-T2F2 is about 1 order of magnitude higher than the values of DPP-T2M2 and DPP-T4.

**Figure 3:**
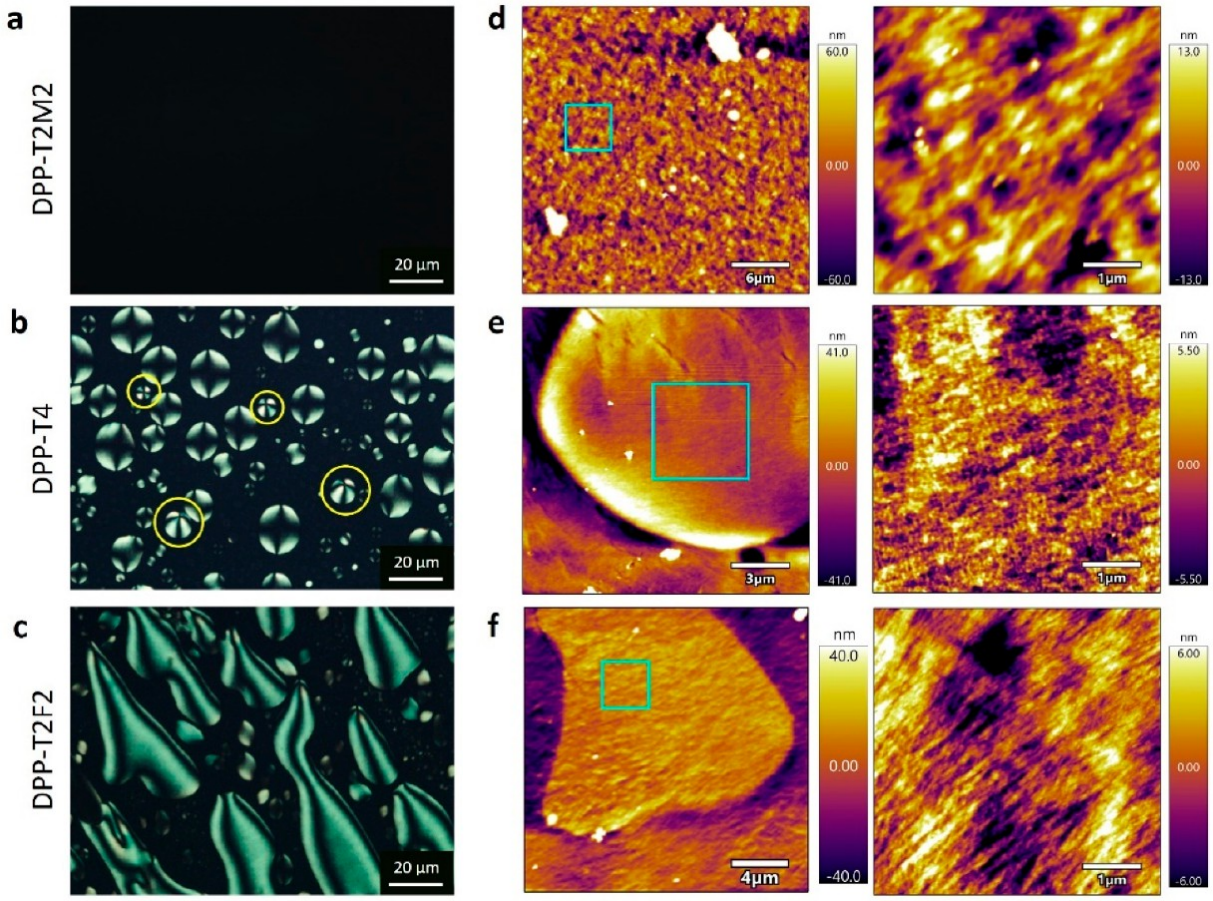
Identification of nanoscale internal structures of chiral twisted-bent mesophases. (a–c) CPOM images of DPP solutions at 100 mg/mL near the equilibrium state, showing the crystalline mesophases for DPP-T4 and DPP-T2F2 whereas isotropic phase for DPP-T2M2. The yellow circles in part b indicate the tactoids with a radial director field. (d–f) AFM images of corresponding 100 mg/mL freeze-dried DPP solutions, displaying nanoscale structures of the mesophases. The right panel shows a magnified view of the selected area (cyan box) in the left panel.

To evaluate how the solution structure defines the emergence of a lyotropic chiral mesophase, we investigated a series of DPP solutions at defined concentrations prepared by a drop-and-dry method. Successive drop-casting from a stock chlorobenzene solution (10 mg/mL) was performed between two glass slides to concentrate solutions up to near 200 mg/mL. The sandwiched solution was then annealed over multiple thermal cycles and equilibrated at room temperature to ensure reaching a near-equilibrium state (see Materials and Methods). The morphology and optical properties of DPP solutions are summarized in Figure 2a, showing that the lyotropic liquid crystals emerge in all DPP systems as the solution concentration increases. In the case of DPP-T2M2, the solution prepared up to ∼100 mg/mL displays no birefringence or apparent aggregates under cross-polarized optical microscopy (CPOM). The solution concentration further increased around ∼160 mg/mL shows a uniform birefringent feature. At an extremely high concentration of ∼200 mg/mL, some alluring texture is observed but difficult to identify under CPOM, which is further explored using SEM and AFM, discussed later. In the case of DPP-T2F2, the LC phase development begins at lower concentration and progresses to more distinct morphologies when compared to the case of DPP-T2M2. Small birefringent microdroplets (tactoids) begin to be shown at ∼60 mg/mL, indicating a transition state where LC mesophases nucleate and grow from an isotropic phase. As the solution concentration increases to ∼100 mg/mL, a uniform morphology with micron-scale domains is observed. The solution at ∼160 mg/mL shows further grown LC mesophase with submicron textures within the domains. At a very high concentration of ∼200 mg/mL, a striped pattern with a few micron periodicity was obtained, which resembles the striped twist-bent mesophase of PII-2T.^18^ In the case of DPP-T4, the mesophase evolution progresses from small LC tactoids at ∼60 mg/mL, larger tactoids at ∼100 mg/mL, and micron-scale LC domains at ∼160 mg/mL to striped textures at ∼200 mg/mL. This DPP-T4 phase development explored under CPOM seems overall analogous to DPP-T2F2 owing to the presence of phases resembling tactoids and striped twist-bent mesophase. The notable difference between DPP-T4 and DPP-T2F2 in this observation, however, is the shape of tactoids and phase transition behavior, which is discussed in detail by characterizing their internal structure of the mesophase, discussed with Figure 3.

Next, considering such a striped texture correlating with chiral mesophases, we used circular dichroism (CD) spectroscopy to explore the chirality of the observed mesophases. Figure 2c–2e shows the CD spectra of DPP solutions at the concentrations corresponding to the CPOM images shown in Figure 2a. The chiroptical property can be described by a dissymmetry factor (*g*-factor), defined as *g* = 2(*A*_L_ – *A*_R_)/(*A*_L_ + *A*_R_), where *A*_R_ and *A*_L_ are right- and left-handed circularly polarized absorbance, respectively. It is basically CD = *A*_L_ – *A*_R_ normalized by the absorbance of nonpolarized light. It is worth noting that the *g*-factor is an intrinsic materials chiral optical property that excludes the explicit dependence on the sample concentration (or density) and path length. Figure 2c shows the chirality emergence of DPP-T2M2 mesophases by an evidence of the increased *g*-factor as the solution concentration increases; zero or negligible CD signal for ≤60 mg/mL, a *g*-factor of 6.2 × 10^–4^, 6.6 × 10^–3^, and −1.0 × 10^–2^ for 100, 160, and 200 mg/mL solution, respectively. Despite the featureless isotropic phase at 100 mg/mL under CPOM, this low but apparent *g*-factor (∼10^–4^) may be associated with molecular and/or nano scale chiral components at this condition. Figure 2d shows CD spectra of DPP-T4 with a *g*-factor of −7.0 × 10^–4^, 3.1 × 10^–3^, −7.2 × 10^–3^, and −8.6 × 10^–3^ for 60, 100, 160, and 200 mg/mL solution, respectively. The chirality development of DPP-T4 mesophases matches the morphological evolution of DPP-T4 crystalline mesophase observed under CPOM. In the case of DPP-T2F2, emerging chirality also follows the morphological evolution of the crystalline mesophase. Figure 2e presents the CD spectra of DPP-T2F2 mesophases with a *g*-factor as follows: 6.1 × 10^–3^, 1.6 × 10^–2^, 3.0 × 10^–2^, and −3.5 × 10^–2^ for 60, 100, 160, and 200 mg/mL, respectively. Interestingly, both DPP-T2M2 and DPP-T2F2 exhibit clear bisignate characters near 750 nm which correspond to the main absorption wavelength of the polymer solution (see Figures S3–S5 for the absorption spectra). This bisignate CD feature presents chiral exciton coupling via Davydov splitting, indicating the electronic or vibrational transition dipoles from polymer backbones are arranged in a chiral fashion.^36^ According to the exciton chirality rule, when the transition dipole moments are arranged in a right-handed fashion, a positive exciton couplet is generated with a positive long-wavelength branch and a negative short-wavelength branch.^36^ The DPP-T2M2 mesophases at 100 and 160 mg/mL show negative and positive CD signs in each long and short wavelength region whose crossover point occurs near 750 nm, indicating the backbones form left-handed helical aggregation. In the case of 200 mg/mL DPP-T2M2, the opposite signs suggest right-handed helical aggregation. Likewise, the handedness of the DPP-T2F2 mesophase is determined by the sign of CD signals. In the case of DPP-T4, no such bisignate features are observed at least in the range of the wavelength we tested except for the CD at the high concentration of 200 mg/mL. This is probably because the origin of CD signals is more related to twisted nano/micrometer structures rather than the polymer backbone. At 200 mg/mL, the backbones may be ultimately torsional and thus associated with the twistedness in the striped twist-bent mesophase. The handedness of DPP-T4 mesophases is simply decided by an overall CD sign; the negative/positive signal indicates left-handed/right-handed (CD = *A*_L_ – *A*_R_). We note that the handedness of each chiral mesophase exhibits stochastic character. Table S3 shows a summary of the handedness probability and the average *g*-factor obtained from a total of 15 samples of each mesophase. The preferential handedness inverts from a twist-bent mesophase to a striped twist-bent mesophase. The origin of this handedness inversion is currently unknown, but similar phenomena is observed in natural cholesteric phases^37^ and PII-2T chiral mesophase as well.^18^ Interestingly, the *g*-factor of the DPP-T2F2 crystalline mesophase at each same solution concentration is consistently higher (approximately 5–10 fold) than those observed in DPP-T2M2 and DPP-T4 (Figure 2f and Table S3). Particularly, the g-factor of DPP-T2F2 at 200 mg/mL even reached up to 0.09, which is a remarkable value for the helical system made of an achiral semiconducting polymer without chiral dopant. A typical *g*-factor of chiral conjugated molecules is around 10^–3^ when their chiral exciton coupling associated in condensed phases.^36^ This high value for DPP-T2F2 is probably due to small helical pitch (shown later) combined with long-range, well-ordered polymer backbones in a helical fashion.^37,38^ Fast Fourier Transform (FFT) images of 200 mg/mL DPP mesophases (Figure 2b) exhibit that the DPP-T2F2 striped twist-bent mesophase forms uniformly over a large area when compared to DPP-T2M2 and DPP-T4. We note the *g*-factor reported in this study is a true value obtained from a genuine CD by ruling out the existence of large LB and LD contributions (see Methods and Figures S3–S5). Thus, it is important to differentiate our values from several macroscopic *g*-factors reported up to ∼0.2 obtained from a pseudo-CD including spurious contributions.^36^ Based on CD and imaging shown later, we name each phase shown in Figure 2a as (i) isotropic phase, (ii) twist-bent mesophase, and (iii) striped twist-bent mesophase. We found later that the aggregates that constitute the mesophase are the same class for DPP-T2M2 and DPP-T2F2 LC mesophases, but different for DPP-T4 mesophases. Also, we confirmed the importance of solution-state aggregation to chiral emergence by investigating how the solvent polarity can affect the solution-state aggregates, and their impact on chiral helical assembly (see Figure S6).

To identify in detail the nano- and micrometer-scale structures formed within the mesophase, we carried out the imaging analysis with AFM and SEM. We employed 100 mg/mL DPP solutions to investigate nanoscale structures that existed in an early stage of each DPP chiral mesophase which exhibit different *g*-factors at the same concentration (Table S3). All solutions were annealed over multiple thermal cycles to ensure reaching a thermodynamic equilibrium state (Methods and Movies S1–S3). Interestingly, in this process, DPP-T4 solution shows a unique phenomenon with increasing temperature, where the aggregates are first wrinkling and then cracking, followed by segregating and dissolving (Movie S2). Figure 3a shows the featureless, dark CPOM image of 100 mg/mL DPP-T2M2 solution, indicating the solution at 100 mg/mL is isotropic at the equilibrium state. In contrast, equilibrium-state DPP-T4 (Figure 3b) and DPP-T2F2 (Figure 3c) show crystalline LC mesophases with each distinct shape of tactoids. We compared the aspect ratio (*L*/*r* = long/short axis) of tactoids by selecting tactoids with a similar solution volume of DPP-T4 (108 ± 42 μL^3^) and DPP-T2F2 (103 ± 40 μL^3^). The DPP-T4 tactoids are close to a spheroidal shape (*L*/*r* = 1.16 ± 0.10), while the DPP-T2F2 tactoids are more elongated to become a spindle shape (*L*/*r* = 1.75 ± 0.28) (Figure S7). The shape of tactoids is determined by a balance between the bulk elasticity and interfacial tension of mesogens, and the volume of the droplet.^39^ Upon comparing the similar volume of tactoids, we attribute the elongated shape for DPP-T2F2 to higher elasticity and/or anisotropic surface tension, while the spheroidal shape for DPP-T4 to the lower elasticity and/or isotropic surface tension.^40^ This difference in the tactoid shapes suggests that nanoscale aggregates comprising the mesophase of DPP-T4 and DPP-T2F2 are very distinct from each other. Figure 3f shows the AFM images of the DPP-T2F2 crystalline mesophase, confirming that fibrillar-shaped aggregates constitute the mesophase. Figure 3e shows the AFM images of DPP-T4 crystalline mesophase. The DPP-T4 nanoscale structure is clearly distinct from that of DPP-T2F2, showing rather a 2D sheet feature with a smooth surface. This observation is consistent with the fact that the DPP-T4 mesophase arises from its 2D sheet aggregates. Moreover, the unique crossed birefringence for DPP-T4 tactoids are observed (yellow circles marked in Figure 3b), confirming the director field is not uniaxial but radial according to previous similar tactoids reported formed by plate-like inorganic particles.^41^Figure 3d shows the AFM images of equilibrium-state DPP-T2M2 at 100 mg/mL. The DPP-T2M2 aggregate structures at micron scale are randomly oriented small domains, however the nanoscale aggregates within these domains show a fibrillar feature.

**Figure 4:**
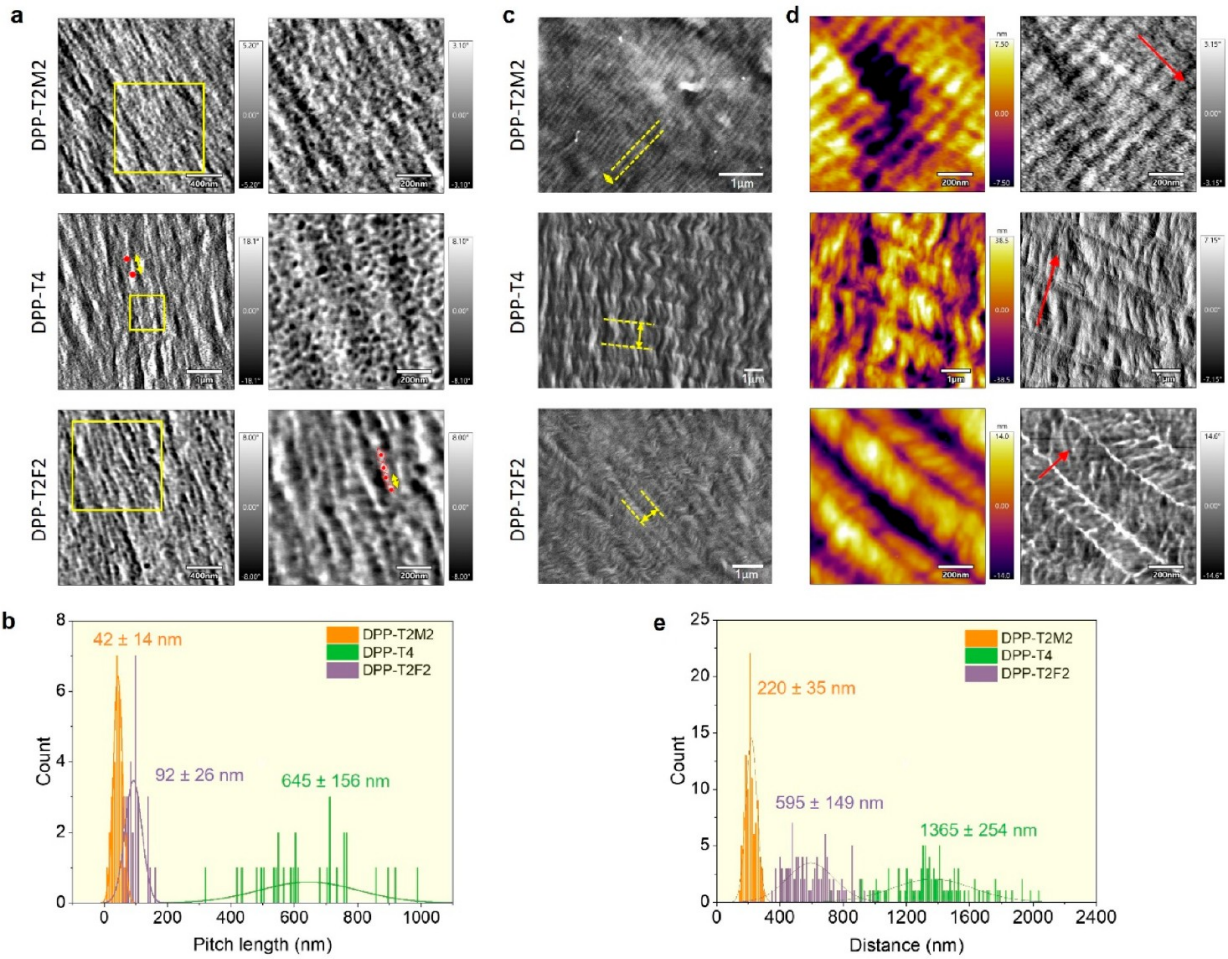
Micron and nanoscale morphology comparison of multiscale DPP helical assembly. (a) AFM phase images of freeze-dried DPP mesophases at ∼160 mg/mL (left). Magnified views are marked with yellow boxes on the left (right). (b) Nanoscale pitch length distribution obtained along the longitudinal axis of the elongated features shown in the AFM images. Examples of measuring the half pitch length are shown with red dots and yellow arrows in AFM images. (c) SEM images of freeze-dried DPP mesophase at ∼200 mg/mL. (d) AFM topography (left) and phase (right) images of the corresponding DPP mesophase shown in c. The red arrows indicate the fiber alignment direction, presumably the polymer chain direction as well. (e) Micron scale pitch length distribution obtained from measuring the distance marked with yellow arrows in c.

When the solution concentration was further increased to 160 mg/mL, we found that the distinct solution characteristic gives rise to distinct helical pitches at two length scales. Figure 4a shows a densely packed morphology composed of highly aligned twisted fibers for DPP-T2M2 and DPP-T2F2. However, DPP-T4 shows an apparent unidirectional twisted morphology, which seems to be “twisted wrinkles” structures rather than “twisted fibers”. Additional image processing and analysis allow us to estimate a helical pitch length of nanoscale twisted features (Figure S8). Figure 4b shows a pitch length distribution of nanoscale twisted features with lengths of 42 ± 14, 92 ± 26, and 645 ± 156 nm for DPP-T2M2, DPP-T2F2 and DPP-T4, respectively. It is noted that the length scale for DPP-T4 is much larger than the other two probably due to higher energetic cost of twisting 2D sheets relative to 1D structures. More interestingly, when the solution concentration further increases to around 200 mg/mL, zigzag twinned morphology is observed for all DPP systems but with each distinct domain length scale (Figure 4c–4d). This unique morphology indicates a striped twist-bent mesophase where densely packed twisted features further twist and bend coherently to result in larger scale zigzag twinned domains.^18^ This feature corresponds to the alternating dark and bright band patterns observed under CPOM for DPP-T4 and DPP-T2M2 (Figure 2a). Figure 4e shows a pitch length distribution of striped twist-bent mesophases with a length of 220 ± 35, 595 ± 149, and 1365 ± 254 nm for DPP-T2M2, DPP-T2F2, and DPP-T4, respectively. A difference between DPP-T4 and DPP-T2F2 was consistently observed–helical, twisted fiber bundles constitute the DPP-T2F2 mesophase, whereas the DPP-T4 mesophase seems to arise from twisting the wrinkles on a 2D sheet. As a result, the length scale of the DPP-T4 twinned domains (1365 ± 254 μm) is thus much larger than the length scale of the DPP-T2F2 domains (595 ± 149 μm). Due to the small pitch (220 ± 35 μm) of the striped twist-bent phase for DPP-T2M2, it is difficult to infer what constitutes the mesophase, but the AFM phase image clearly shows fibrillar features (zoom-in images in Figure S9).

**Figure 5:**
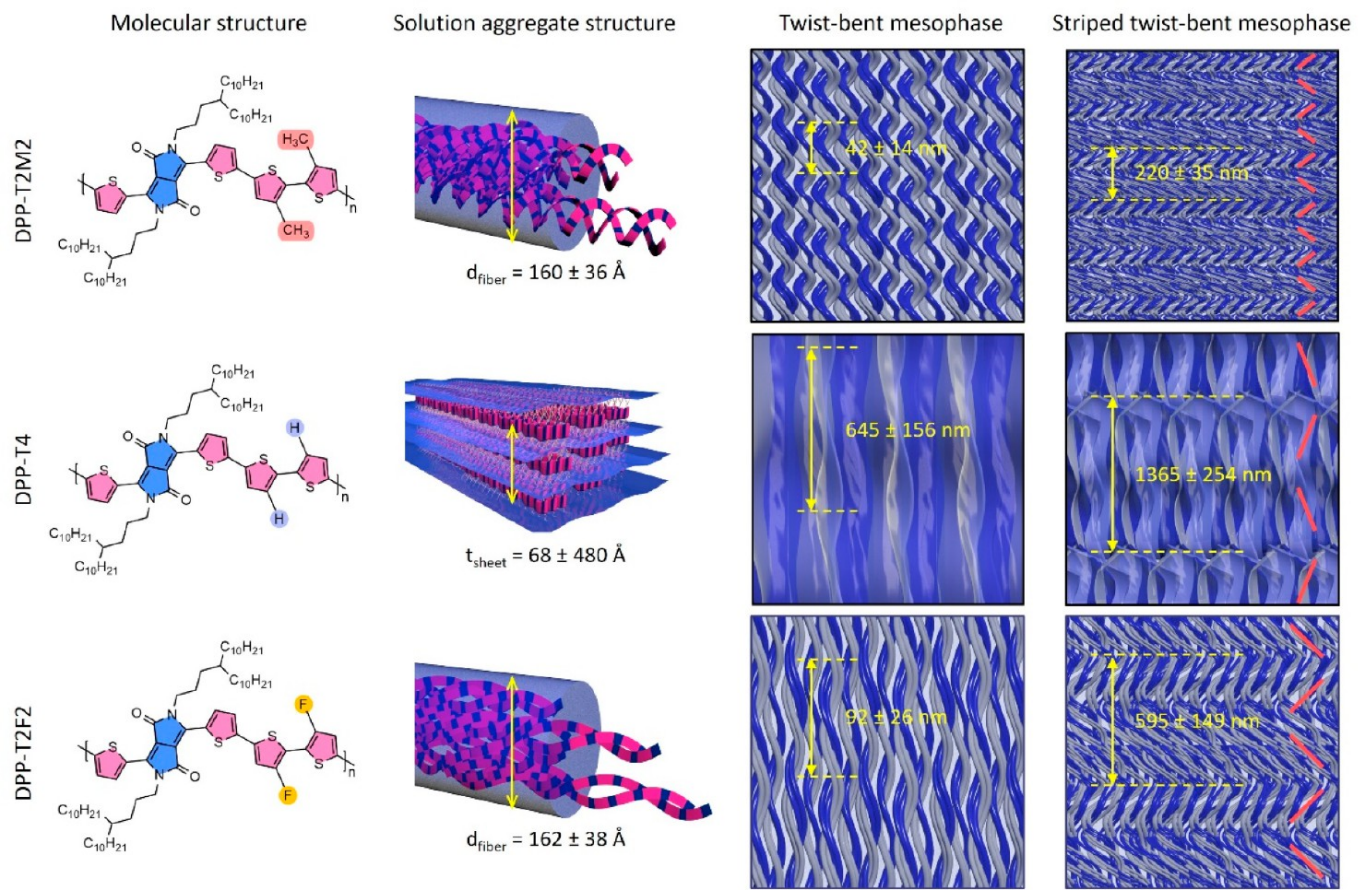
Schematic illustration of proposed chiral evolution in multiscale hierarchical assembly of achiral DPP conjugated polymers. DPP-T2M2 and DPP-T2F2 form helical nanofibers with different pitch lengths due to the difference in their polymer chain flexibility. On the other hand, DPP-T4 produces thin 2D sheet aggregates facilitated through backbone and lamellar stackings. Increasing the concentration leads the helical nanofibers and thin 2D sheets to assemble into a coherent twist-bent mesophase and eventually a striped twist-bent mesophase. The red lines in striped twist-bent mesophase indicate the zigzag morphologies.

Figure 5 illustrates proposed assembly mechanisms of how each distinct solution-state aggregate evolves multiscale helical assemblies of achiral DPP conjugated polymers. Three DPP systems that we studied are designed to tune the backbone torsion by replacing the hydrogen atoms with fluorine atoms or methyl groups on T-T units. We found that these three polymers reveal distinct solution-state aggregate structures in dilute solutions: semicrystalline 1D helical fiber aggregates for DPP-T2M2 and DPP-T2F2, and crystalline 2D sheet aggregate for DPP-T4. When increasing solution concentration beyond a threshold volume fraction, dispersed nanoscale aggregates form a lyotropic twist-bent mesophase. Intriguingly, what constitutes the DPP-T4 twist-bent mesophase is “twisted wrinkles” structures, while helical, twisted fiber bundles constitute the DPP-T2M2 and DPP-T2F2 twist bent mesophase. Further increasing concentration induces striped twist-bent mesophase where densely packed fibers twist and bend coherently to result in zigzag twinned domains. In this zigzag twinned morphology, the long-axis of DPP-T2M2 and DPP-T2F2 fibers (polymer chains) and also the long-axis of wrinkles in DPP-T4 (marked with red arrows in Figure 4d) are oriented perpendicular to the twin domain grain boundary. We consistently observed substantially larger helical pitch in DPP-T4 than in DPP-T2M2 and DPP-T2F2 in both the twist-bent and striped twist-bent mesophases. We further observe a correlation between the larger helical pitch observed in the stripped twist-bent mesophase and the smaller helical pitch observed in the twist-bent mesophase before twinned domains emerge (Figure 5). The larger pitch is approximately 5–6 folds that of the smaller pitch for DPP-T2M2 and DPP-T2F2, in contrast to ∼2-fold difference for DPP-T4. It seems the two adjacent “wrinkled” helical structure is simply folded, showing about 2-fold larger length in the twinned morphology for DPP-T4. This distinction, together with the significantly larger helical pitch, collectively points to the uniqueness of DPP-T4 chiral emergence due to its distinct 2D aggregate structure from the 1D aggregates of the other two polymers.

**Figure 6:**
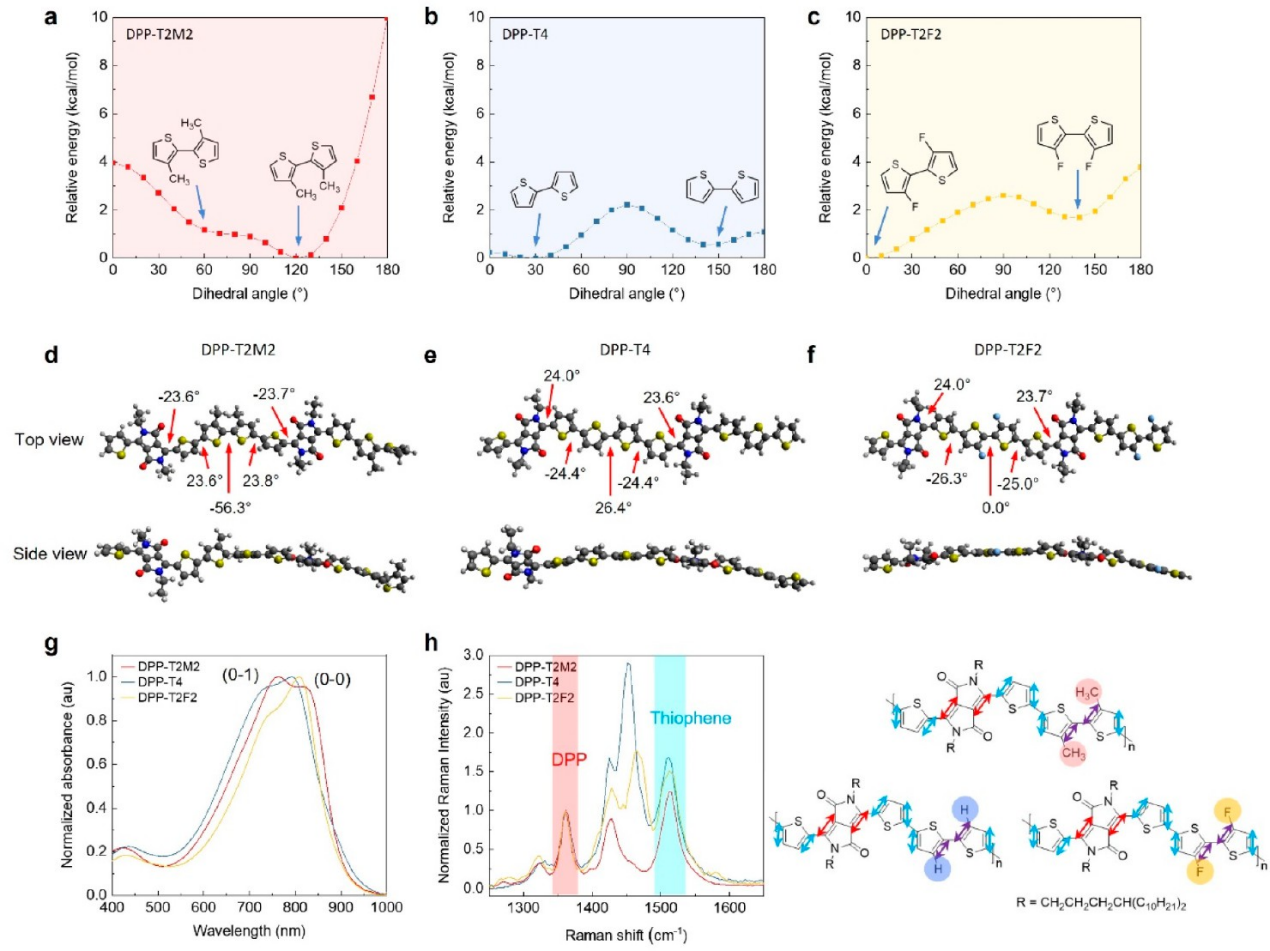
A comparison of DPP molecular conformation. (a–c) Potential energy plots for the dihedral angle between two functional thiophenes for each DPP system. The dihedral angle of 0° refers to an anti-conformation in which sulfur atoms on adjacent thiophenes face the opposite direction, while the angle of 180° refers to a syn-conformation with sulfur atoms faced on the same side. The molecular fragments used for obtaining the plots are inset in the panel. (d–f) Top and side views of optimized dimers of DPP systems with average dihedral angles. The side view of energy-minimized conformers depicts the varying degrees of backbone coplanarity. (g) UV–vis absorption spectra of DPP solutions at 10 mg/mL, clearly showing vibronic characters assigned as (0–0) and (0–1) around 820 and 725 nm, respectively. (h) Raman spectra of freeze-dried isotropic DPP solutions at 10 mg/mL (left) and illustration of representative Raman-active vibrational modes (right). The peak intensity is normalized by the DPP peak around 1360 cm^–1^ (red shade), which is noted by the red arrows in the illustration. The peak around 1520 cm^–1^ (cyan shade) is the delocalized C=C stretching over thiophene units, illustrated with the cyan arrows in the molecular structures.

We further probe into the molecular origin of such distinct solution aggregation behavior and the difference in chiral helical structures by characterizing the molecular confirmation through DFT calculations, UV–vis absorption, and Raman spectroscopy. First, DFT calculations were performed by using the method of ωB97xD/6-31G (d, p) with dispersion correction which is more accurate in estimating the torsional barrier height and dihedral angle of conjugated polymers.^42,43^Figure 6a–6c show potential energy plots corresponding to the T-T dihedral angle between each characteristic thiophene: methylated thiophenes for DPP-T2M2, thiophenes for DPP-T4, and fluorinated thiophenes for DPP-T2F2. We focus our discussion on comparing these dihedral angles because the potential energy scans of dihedral angles between donor and acceptor moieties and between two adjacent thiophenes close to the DPP unit were found to be similar for all DPP systems (Figure S10). Each plot shows two local minima corresponding to the dihedral angles of anti- and syn-conformations. The dihedral angle close to 0° refers to an anti-conformation in which sulfur atoms on adjacent thiophenes face the opposite direction while the angle close to 180° indicates a syn-conformation with sulfur atoms faced on the same side. We found that the optimum DPP-T2F2 is a planar anti-conformation, resulting from its high torsional barrier as well as its lowest relative energy at 0° (Figure 6c and 6f). The fluorine has little steric hindrance and provides attractive noncovalent interactions with adjacent sulfurs, which plays a significant role in promoting the planarization of DPP-T2F2. Also, the strong F···S interaction at the anti-conformation leads to a relatively steep and high torsional barrier and only one predominant conformation at 0°. In contrast, DPP-T2M2 tends to form a highly torsional conformation with an angle of ∼60° due to the strong steric hindrance from the methyl group (Figure 6a and 6d). Interestingly, the small energy barrier between the anti- and syn-conformation likely causes DPP-T2M2 to have more than one conformation in coexistence. In the case of DPP-T4, the global energy minimum is at ∼30°; however, given an almost flat energy profile from 0° to 30°, the T-T dihedral will likely be flexible within this range (Figure 6b and 6e). Further, there is a local minimum at 140° corresponding to the syn-conformation that also has a relative flat energy profile from 140° to 180°. We anticipate that such flexible T-T dihedral in both anti- and syn-conformation offers the degree of freedom for the DPP-T4 backbone to easily adapt to highly crystalline ordering during the 2D sheet formation.

Complementing the DFT calculations, we characterized the DPP molecular conformations in solution by UV–vis absorption and Raman spectroscopy. Figure 6g shows UV–vis absorption spectra of isotropic DPP solutions at a low concentration of 10 mg/mL. The two peaks around 820 and 725 nm clearly indicate a vibronic character given the difference of 1400 cm^–1^; this particular number corresponds to the vibrational frequency of the aromatic-quinoidal stretching mode for nearly all π-conjugated systems.^44^ Therefore, the peak at 820 nm can be assigned as the ground-state electronic transition (0–0) and the 725 nm peak is its higher order vibronic transition, (0–1). The relative intensity of the vibronic progression characterized by the absorption peak ratio (0–0)/(0–1) has been used to indicate conjugation length and polymer conformation.^44^ The ratio is 0.95, 1.05, and 1.20 for DPP-T2M2, DPP-T4, and DPP-T2F2 solution, respectively. This suggests that DPP-T2M2 is more flexible or has higher backbone torsional angles, leading to an increased conformational disorder and decreased effective conjugation length. DPP-T2F2 is a contrary scenario, being relatively more rigid and planar with a decrease in backbone torsion and increase in π-conjugation. The conjugation length of DPP-T4 likely sits in between DPP-T2M2 and DPP-T2F2. Furthermore, we observed that kinetics of solution aggregates goes as DPP-T2M2 < DPP-T4 < DPP-T2F2 (Figure S11), which is directly related to their propensity to form ordered solution state aggregates. Raman spectroscopy was used to confirm the polymer conformational difference by examining the π-electron density distribution in the polymer backbone. Figure 6h shows Raman spectra of freeze-dried DPP solutions at 10 mg/mL. The peak intensity is normalized by the DPP peak around 1360 cm^–1^ by assuming that its fused rings are little influenced by the far-positioned functional thiophenes. Figure 6h (right) shows representative Raman-active vibrational modes for each DPP polymer, determined by other theoretical and experimental studies.^45^ The peak around 1360 cm^–1^ is assigned as strong localized C=C stretching in the DPP units. The peak around 1520 cm^–1^ is assigned as delocalized C=C stretching over the thiophene rings. These peaks show no significant changes in mode frequency, which indicates that the rest of the molecular structure in the conjugated backbone is nearly unaffected by the substitution. The peaks around 1420 and 1460 cm^–1^ are the most different in the position and intensity, which are attributed to the localized carbon bond stretching of the functionalized thiophene units. Because the Raman scattering intensities in conjugated polymers arise from the polarizability of the π-electrons, the peak intensity change indicates a π-electron density on the functional thiophene ring unit, relative to the DPP unit. In the case of DPP-T2M2, the lowest peak intensity at 1420 cm^–1^ and even the almost absent peak at 1460 cm^–1^ indicate that the polymer chains exist as the most twisted/disordered conformation. In contrast, DPP-T4 shows the highest peak intensity among the three DPP systems, meaning the relatively planar conformation with an increase in effective conjugation length. In the case of DPP-T2F2, the low peak intensity and notable peak shift to higher frequency are observed, which is consistent with the previous studies when fluorine substitution increases the coplanarity of the polymer backbone.^46,47^ While a typical backbone planarization is accompanied by a shift of the C=C stretching to lower frequency due to an increase in the bond lengths, this peak shift to higher frequency suggests that the fluorine substitution causes a reduction in the C = C bond lengths, presumably due to the strong electron withdrawing effect of the fluorine atoms. When we compare the Raman spectra for chiral DPP mesophases at high concentrations, all thiophene peak intensities relative to the DPP unit decrease as the molecular conformation twists more (Figure S12). It is noted that the extent of the intensity changes for each DPP mesophase relative to their isotropic phase is different; the lowest change for DPP-T2M2, and more drastic changes for DPP-T2F2 and DPP-T4. Because the DPP-T2M2 molecular conformation is already highly torsional at low concentrations according to UV–vis, while it becomes more torsional as chiral mesophases emerge at higher concentrations, the extent of change in torsional angle is relatively small. On the other hand, DPP-T2F2 is highly planar at low concentration due to the conformation lock, which is then broken at higher concentrations to result in highly torsional backbone when chiral mesophases emerge. The originally planar DPP-T4 also becomes highly torsional at high concentrations, possibly owing to the flexible T-T dihedrals according to DFT calculations. The concentration-induced increase in backbone torsion is consistent with the emergence of chiral mesophases across the three polymers. On the other hand, there are important distinctions that help rationalize the difference in chiral helical structures among three polymers. In contrast to the other two polymers, DPP-T4’s T-T dihedrals are very flexible, so they can easily adapt to crystallization. This character results in 2D crystalline sheet-like aggregates in solution and twisted wrinkles on 2D sheets with a large pitch length. While both DPP-T2M2 and DPP-T2F2 mesophases are composed of 1D fibers, their helical pitch length difference in the twist-bent LCs can be explained by their torsion angles. Although the originally planar DPP-T2F2 becomes torsional at high concentrations (when the mesophases emerge), it is still less torsional than DPP-T2M2, according to the concentration dependent UV–vis absorption spectra shown in Figure S12a. Hence, the more torsional backbone of DPP-T2M2 gives rise to a shorter pitch length of chiral helical structures than DPP-T2F2.

## Conclusions

In summary, we present distinct solution-state aggregate structures and their impact on chiral helical assemblies using three DPP systems with subtle changes in thiophene substitution designed to vary backbone torsions. Through a combination of X-ray scattering and imaging analysis, we find that DPP-T2M2 and DPP-T2F2 form semicrystalline 1D fiber aggregate with distinct internal structures and fiber rigidity, whereas DPP-T4 produces crystalline 2D sheet aggregates in solution owing to its highly flexible T-T dihedral. Moreover, we observe that each DPP aggregate assembles into chiral LC mesophases as the fibers and sheets become more twisted and bent in a helical fashion. DPP-T2M2 and DPP-T2F2 fall into the same class by following the helical nanofiber mediated assembly pathway with an increasing concentration. On the other hand, the assembly mechanism of DPP-T4 seems to be distinct given its unique 2D sheet solution aggregate structure. DFT calculations and optical spectroscopy analysis suggest that highly flexible T-T dihedrals for DPP-T4 can allow the polymer chains to easily adapt to crystallization, resulting in 2D crystalline sheet aggregates in solution. Thus, the nano/micron helical pitch lengths for DPP-T4 are much larger than the other two polymer systems probably due to the higher energetic cost of twisting 2D sheets relative to 1D fibers. Comparing DPP-T2M2 and DPP-T2F2, the difference between their helical pitch possibly originates from their distinct backbone conformation: more torsional DPP-T2M2 stack loosely to form more flexible 1D fibers and result in shorter helical pitch, whereas more planar DPP-T2F2 forms rigid 1D fibers with more ordered internal packing to yield longer helical pitch and long-range order. Combining a relatively small helical pitch with exceptional long-range order, DPP-T2F2 yielded an anisotropic dissymmetry factor (g-factor) as high as 0.09. Our findings overall raise the point that “Not all aggregates are made the same”^14^—it is critical to not overlook the solution structure to decipher complex hierarchical mesophases and to discover even unknown phases that can further lead to optical, electronic, and mechanical properties unimagined before.

## Supporting Information Available

Materials and methods for polymer synthesis and mesophase samples, experimental details and analysis, as well as additional figures and tables (PDF)
In-situ cross polarized optical microscopy (CPOM) movie of a 100 mg/mL DPP-T2M2 polymer solution monitored during thermal heating and cooling (AVI)
In-situ cross polarized optical microscopy (CPOM) movie of a 100 mg/mL DPP-T4 polymer solution monitored during thermal heating and cooling (AVI)
In-situ cross polarized optical microscopy (CPOM) movie of a 100 mg/mL DPP-T2F2 polymer solution monitored during thermal heating and cooling (AVI)
SAXS model (ZIP)

## Supplementary Material

oc3c00775_si_001.pdf

oc3c00775_si_002.avi

oc3c00775_si_003.avi

oc3c00775_si_004.avi
