# Supplementary material for: Subtle Molecular Changes Largely Modulate Chiral Helical Assemblies of Achiral Conjugated Polymers by Tuning Solution-State Aggregation
Source: ACS Cent Sci. 2023 Nov 13;9(11):2096–107. doi: 10.1021/acscentsci.3c00775 (PMC10683494; doi:10.1021/acscentsci.3c00775)
Supplement: Supplementary file 1 — oc3c00775_si_001.pdf [file oc3c00775_si_001.pdf]

Supporting Information

Subtle molecular changes largely modulate chiral helical assemblies of achiral conjugated polymers by tuning solution-state aggregation

Kyung Sun Park[1], Xuyi Luo[2], Justin J. Kwok[3], Azzaya Khasbaatar[1], Jianguo Mei[2] and Ying Diao[1,3,4,5,6*]

[1]Department of Chemical and Biomolecular Engineering, University of Illinois at Urbana-Champaign, 600 S. Mathews Ave., Urbana, IL 61801, USA.

[2]Department of Chemistry, Purdue University, 560 Oval Dr., West Lafayette, IN 47907, USA.

[3]Department of Materials Science and Engineering, University of Illinois at Urbana-Champaign, 1304 W. Green St., Urbana, IL 61801, USA.

[4]Beckman Institute, Molecular Science and Engineering, University of Illinois at Urbana-Champaign, 405 N. Mathews Ave., Urbana, IL 61801, USA.

[5]Department of Chemistry, University of Illinois at Urbana-Champaign, 505 S. Mathews Ave., Urbana, IL 61801, USA.

[6]Materials Research Laboratory, The Grainger College of Engineering, University of Illinois at Urbana-Champaign, 104 S. Goodwin Ave., Urbana, IL 61801, USA.

*Corresponding author. Email: yingdiao@illinois.edu

Materials and methods for synthesis of DPP-T2M2, DPP-T4 and DPP-T2F2

DPP-4T, DPP-T2M2 and DPP-T2F2 were synthesized via Stille polymerization from the DPP-2T monomer and the corresponding bithiophene ditin compounds.

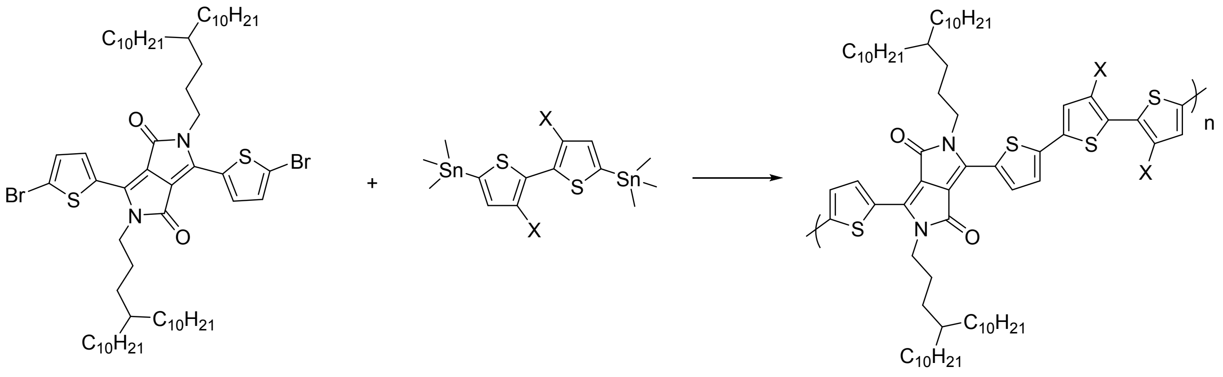

DPP-310

X = H, T2
X = CH_3, T2M2
X = F, T2F2

X = H, DPP-T4
X = CH_3, DPP-T2M2
X = F, DPP-T2F2

First, DPP-310 (1.0 equiv.), ditin Compound T2, T2M2 or T2F2 (1.0 equiv.), Pd2(dba)3 (2.5 mol%), P(o-tol)3 (4 mol%) and 20 mL of toluene were added in a Schlenk tube charged with a stir bar. The resulting solution was bubbled with argon for 20 min, and the mixture was stirred at 110 °C for 48 h. After completed the polymerization, the mixture was taken up and precipitated into methanol. The polymer solid was collected by a Soxhlet thimble, which was purified by Soxhlet extraction successively with methanol, acetone, hexane, then collected by chloroform. To remove the residual catalyst, N,N-diethyl-2-phenyldiazenecarbothioamide was added to the chloroform fraction, and the mixture was stirred at 60 °C for 30 min before being precipitated into methanol. The collected polymer was dried at 60 °C under vacuum. The yield and the molecular weight which evaluated by Size Exclusion Chromatography are summarized as follows:

DPP-T4: 150.0 mg (0.1326 mmol) of DPP-310 and 65.21 mg of (0.1326 mmol) T2 were used. Precipitation provided 117 mg (78% yield) of a dark solid. Mn = 12.2 kDa, Mw = 25.7 kDa PDI = 2.1.

DPP-T2M2: 150.0 mg (0.1326 mmol) of DPP-310 and 68.93 mg (0.1326 mmol) of T2M2 were used. Precipitation provided 131 mg (85% yield) of a dark solid. Mn = 30.7 kDa, Mw = 68.1 kDa, PDI = 2.22.

DPP-T2F2: 150.0 mg (0.1326 mmol) of DPP-310, 69.98 mg (0.1326 mmol) of T2F2 were used. Precipitation provided 140 mg (90% yield) of a dark solid. Mn = 21.8 kDa, Mw = 122.6 kDa, PDI = 5.63.

Materials and Methods

The DPP polymer solutions were prepared by dissolving the polymer (10 mg/ml) in chlorobenzene (CB; anhydrous, 99.8%; Sigma-Aldrich Inc.). Silicon (0.001-0.005 Ohm.cm, Namkang Hightech), micro slide glass (VWR Cat No 48366-067) and corning glass substrates (Fair & Cheer Inc.) were used to prepare sandwiched solutions. The substrates were cleaned with toluene, acetone, and isopropyl alcohol and then blow-dried with a stream of nitrogen to remove contaminants. Poly(sodium 4-styrenesulfonate) (PSS) average Mw ~70,000, powder (Sigma-Aldrich Inc.) was used as a sacrificial layer to transfer the polymer films for TEM characterization.

SAXS experiments and analysis

SAXS experiments were carried out at the 12-ID-B beamline of the Advanced Photon Source at Argonne National Laboratory using an X-ray beam energy of 13.3 keV. A Pilatus 2M detector was used primarily at a sample-to-detector distance of 3.6 m. The polymer solution SAXS experiments were performed using a flow cell to prevent beam damage and enable longer exposure times[S1]. The flow cell was constructed using a 1 mm diameter quartz capillary connected to PTFE tubing using PTFE heat shrink tubing. The tubing was connected to a syringe pump which cycled the polymer solution at a linear velocity of about 1 mm/s within the capillary while a series of 0.1 s exposures with 3 s delays were accumulated. The isotropic 2D scattering patterns were averaged, reduced, and then background subtracted using the beamline's MATLAB package. The 1D scattering profiles were then analyzed and fitted using custom models in SasView.

Solution-state sample preparation and characterization

All solution samples except the ones for SAXS were prepared by a drop-and-dry method[S2]. The drop-and-dry method is basically concentrating the pristine solution by adding multiple numbers of solution droplets, drying them out and blending with a solution drop. Briefly, a needed concentration was obtained from casting and drying a multiple number of the solution drops on a local spot of the substrate first and blending/shearing with the last drop of the stock solution (2 μl). The sandwiched solution samples were further run through thermal annealing cycles to reach an equilibrium state. For instance, a 60 mg/ml solution was made with dried five drops of 2 μL 10 mg/ml solution on a substrate and subsequently blending by additional one drop of 2 μL 10 mg/ml solution with a glass coverslip. In order to reach an equilibrium

state, the sandwiched sample was run through a moderate heating and cooling process (25 °C→100 °C→25 °C) on a Linkam thermal stage (LTS420). The rate of heating and cooling was 5 °C/min. The sandwiched solution was annealed over multiple thermal cycles (2-4 cycles) and equilibrated at room temperature to ensure reaching equilibrium state. The birefringence of mesophases was observed using CPOM (Eclipse Ci-POL, Nikon). UV-vis (Cary 60 UV-Vis, Agilent) spectroscopy was used to investigate polymer conformation in the solution phase. CD spectra were recorded using a JASCO J-1500 spectrophotometer. To eliminate contributions from linear dichroism and birefringence, all samples were investigated by averaging four measurements with 90° in-plane rotation and 180° out-of-plane rotation of the sample[S3,S4]. Identical spectra were recorded at different sample batches demonstrating the chiral nature of the phase.

Freeze-drying solution samples and characterization

The solution-state samples sandwiched between a silicon or PSS coated silicon substrate and a glass coverslip were first submerged in a liquid mixture of 63% propane and 37% ethane for about a minute and then stored in liquid nitrogen. Since this mixture has a larger heat capacity than liquid nitrogen, it can quickly freeze the sample without boiling off and therefore prevent aggregation during the cooling process [5]. The top glass coverslip was then removed inside the liquid nitrogen bath. The sample was immediately transferred to a sealed Linkam thermal stage chamber (LTS420) which is held at -100 °C in a nitrogen atmosphere. The temperature was slowly (0.5 °C/min) raised to -80 °C for chlorobenzene sublimation under the vacuum. It took ~6 hours to fully sublimate the solvent. Finally, the temperature was raised to 25 °C. The prepared samples on silicon substrates were directly imaged by using SEM (Hitachi 4800) at 25 kV or 3 kV accelerating voltage and AFM (Asylum Research Cypher, tapping mode) with silicon AFM probes (Ted Pella, Tap 300 AL -G). Additional metal coating was avoided when SEM measurements to preserve the original surface morphology of the polymer samples. For TEM imaging (JEOL 2100 cryo TEM with a LaB6 emitter at 200 kV), low electron dose rates (4–12 e^{-} $Å^{-2}$ s^{-1}) were applied using spot size 3 to minimize beam-induced alteration. Each image was collected with an exposure time of 1 s, resulting in a dose per image of 4–12 $e^{-}Å^{-2}$. TEM samples were prepared the same as described above but performed on PSS layer deposited silicon substrates. 10 wt% PSS in water solution was spin coated on the silicon substrate at 5000 rpm for 1 min. Once freeze-drying polymer films prepared on the PSS were submerged in a water bath, thin films were immediately floating on the top surface of water as

the PSS was dissolved in water. Then, the thin films were carefully transferred on copper grids (Ted Pella, 01840-F). The molecular conformation was analyzed using a Raman confocal imaging microscope (LabRAM HR 3D-capable Raman spectroscopy imaging system, Horiba) with ×100 objective lens equipped with a Horiba Synapse back-illuminated deep-depletion charge-coupled device camera. A 532-nm laser (maximum power, 50 mW; Laser Quantum) was used as the excitation source. Integration times of 10 s were used for each measurement. An optical density (OD) filter = 1 was used [OD = log (power transmission factor)] after confirming that there was no beam damage to the sample.

Density Functional Theory Calculations

DFT calculations were used for molecular geometry optimization and obtaining potential energy scans of the dihedral angles. All calculations were performed with the Gaussian 16 package by using wB97xD method at 6-31G (d, p) basis set. In these calculations, the long alkyl sidechains were replaced with methyl groups for simplification. To obtain the potential energy scans of a dihedral angle, each monomer was first optimized at the wB97xD/6-31G (d, p) level of theory. Afterwards, a rigid scan where the single point energy of the optimized molecule was computed when the dihedral angle of interest was varied from 0° to 180° at 10° interval while fixing all other dihedrals. The resulting single point energies were then plotted against the dihedral angle to obtain the potential energy plots.

Table S1. Fitting parameters for the 2SFC model used for DPP-T2M2 and DPP-T2F2.

Sample	L_{fiber} (nm)	b_{fiber} (Å)	R_{fiber} (Å)	L_{chain} (nm)	$\boldsymbol{l_{chain}}$ (Å)	$R_{polymer}$ (Å)	q_c	w	μ
T2M2	1000*	27.7 ± 36.4	80.4 ± 18.3	500*	~10^{7}	29.8 ± 18.4	0.21 ± 0.09	0.23 ± 0.29	1
T2F2	1000*	6.4 ± 6.5	81.4 ± 19.9	500*	~10^{14}	16.2 ± 17.0	0.20 ± 0.16	0.19 ± 0.24	1

*Values were fixed during fitting.

L-contour length; b-Kuhn length; R-radius; *l*-persistence length; q_c-peak center of pseudo-Voigt peak function; w-full width at half-maximum of pseudo-Voigt peak function; μ-Lorentzian fraction of pseudo-Voigt peak function.

Table S2. Fitting parameters for the SA model used for DPP-T4.

	a_{sheet} (nm)	b_{heets} (nm)	c_{sheet} (Å)	q_c (s1)	w (s1)	μ (s1)	q_c (s2)	w (s2)	μ (s2)	q_c (br)	w (br)	μ (br)
T4	1000*	1000*	68.8 ± 479.7	0.19 ± 0.041	0.023 ± 0.18	1	0.38 ± 0.61	0.032 ± 2.0	1	0.18 ± 0.33	0.25 ± 2.1	1

*Values were fixed during fitting.

a,b,c-width, length and thickness, respectively of the parallelepiped; q_c-peak center of pseudo-Voigt peak function; w-full width at half-maximum of pseudo-Voigt peak function; μ-Lorentzian fraction of pseudo-Voigt peak function; s1-sharp peak 1; s2-sharp peak 2; br-broad peak.

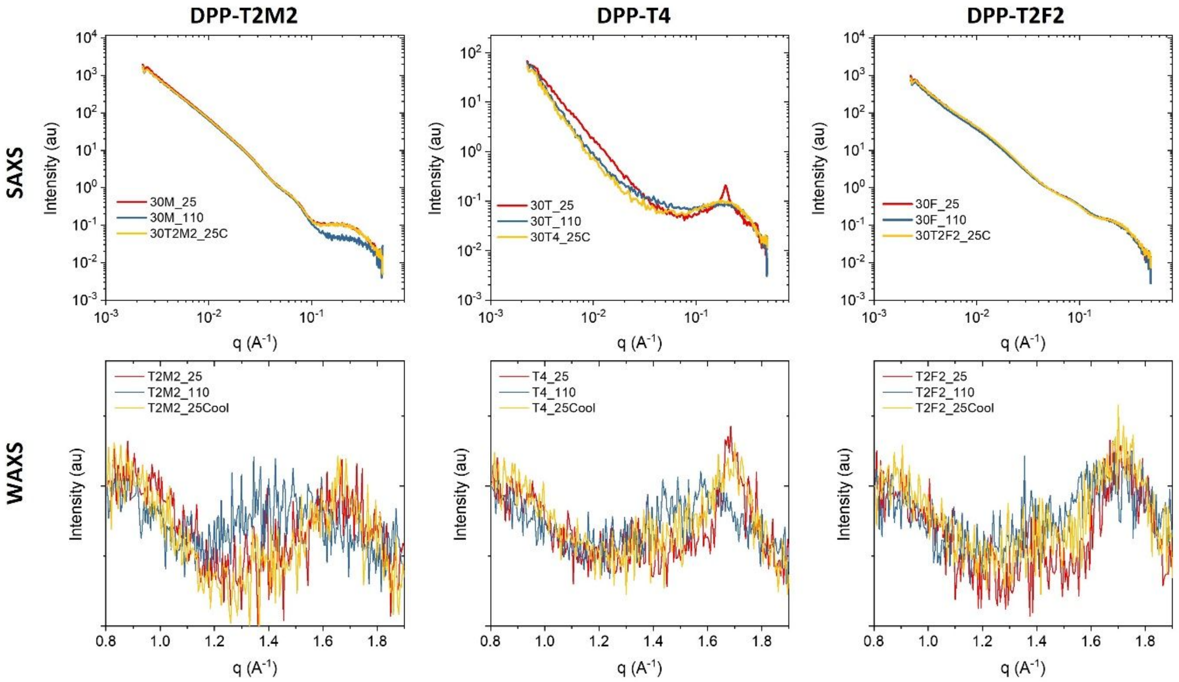

Figure S1. In-situ solution X-ray scattering by thermally annealing the solution at 110 °C and then cooling the solution at 25 °C. The slope at low q for DPP-T4 is changed from 2.7 to 3.4 upon heating and cooling. It is noted that the irreversible crystalline aggregates are probably due to the slow kinetic in the given measuring time (approximately 10 min).

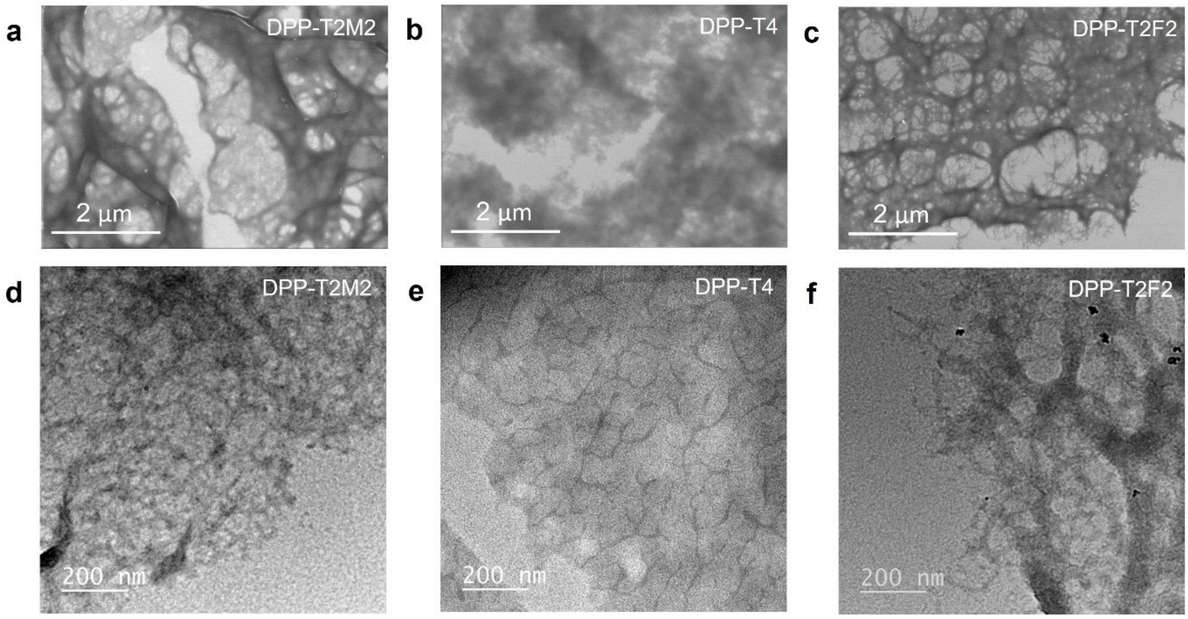

Figure S2. (a-c) SEM and (d-f) TEM images of freeze-dried samples prepared from the solution at the same concentration taken by SAXS.

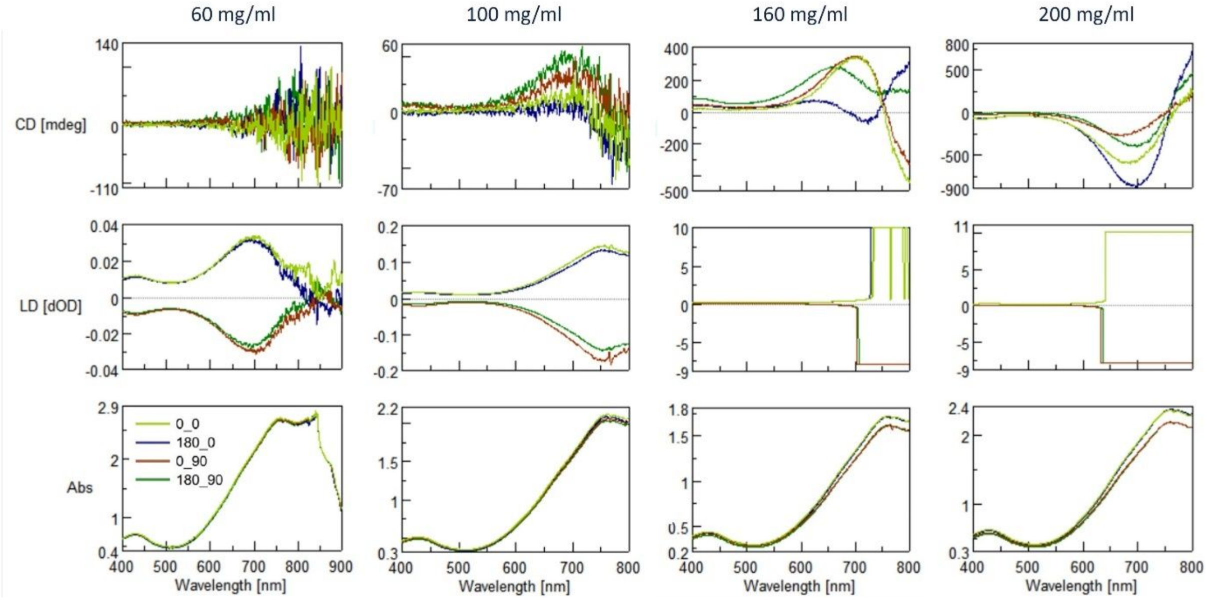

Figure S3. CD, LD and absorbance (Abs) of DPP-T2M2 solutions at each concentration denoted. The four measurements by in-plane rotating of 90° and out-of-plane rotating of 180° (flipping) were performed to obtain a true CD by excluding any linear birefringence (LB)/ linear dichroism (LD) contributions. According to the most common theoretical model used to account the CD signal in anisotropic samples [Ref S4], four measurements were conducted: averaging two spectra obtained by flipping 180° out-of-plane rotation can cancel the LB/LD contribution, whereas averaging two spectra obtained by 90° in-plane rotation can lead to the cancellation of the LD contribution. The CD signals shown in the manuscript were obtained by following a careful method to exclude any artifacts arising from LD and LB contributions.

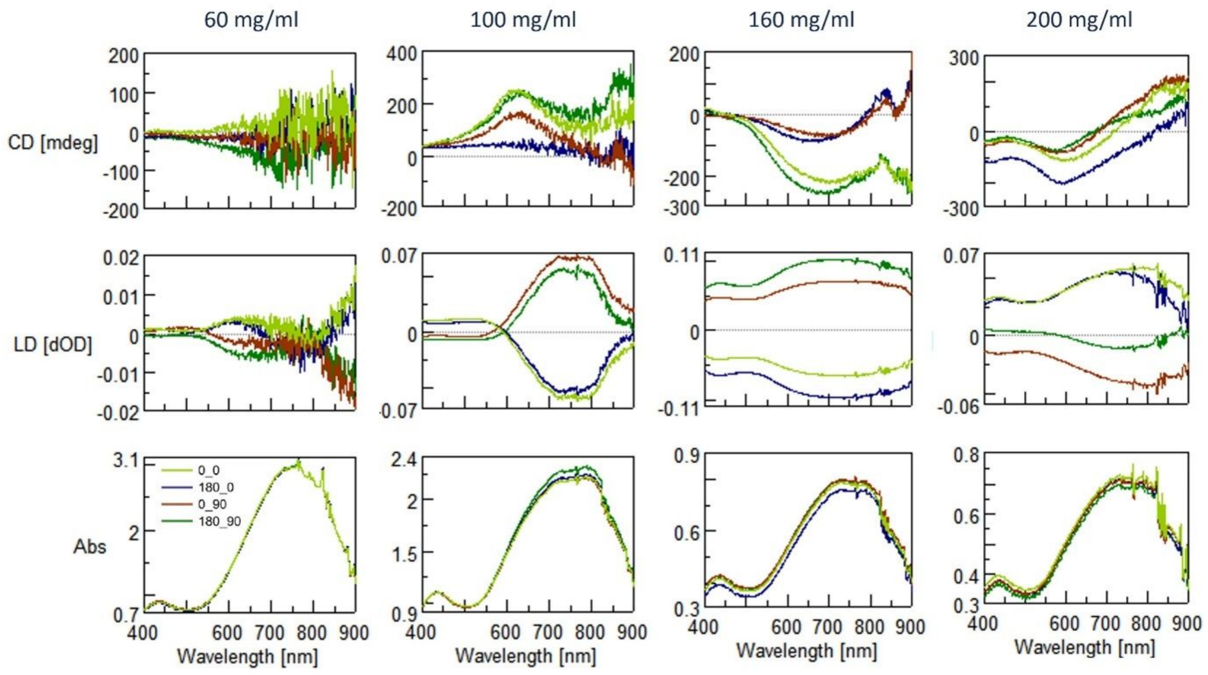

Figure S4. CD, LD and Absorbance of DPP-T4 solutions at each concentration denoted. The same four measurements were performed.

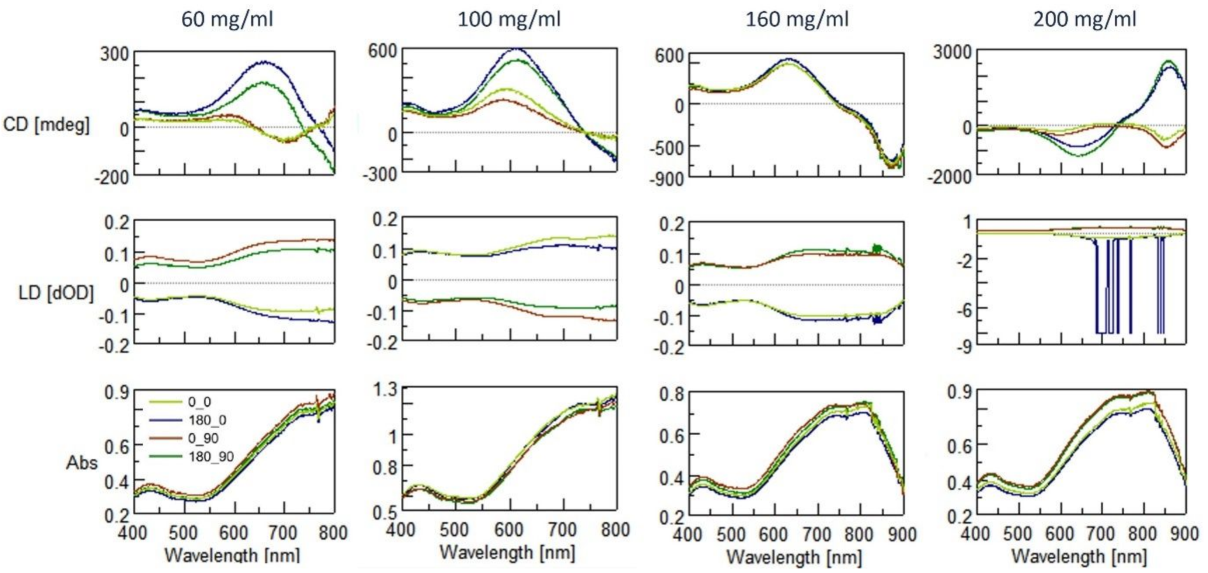

Figure S5. CD, LD and Absorbance of DPP-T2F2 solutions at each concentration denoted. The same four measurements were performed.

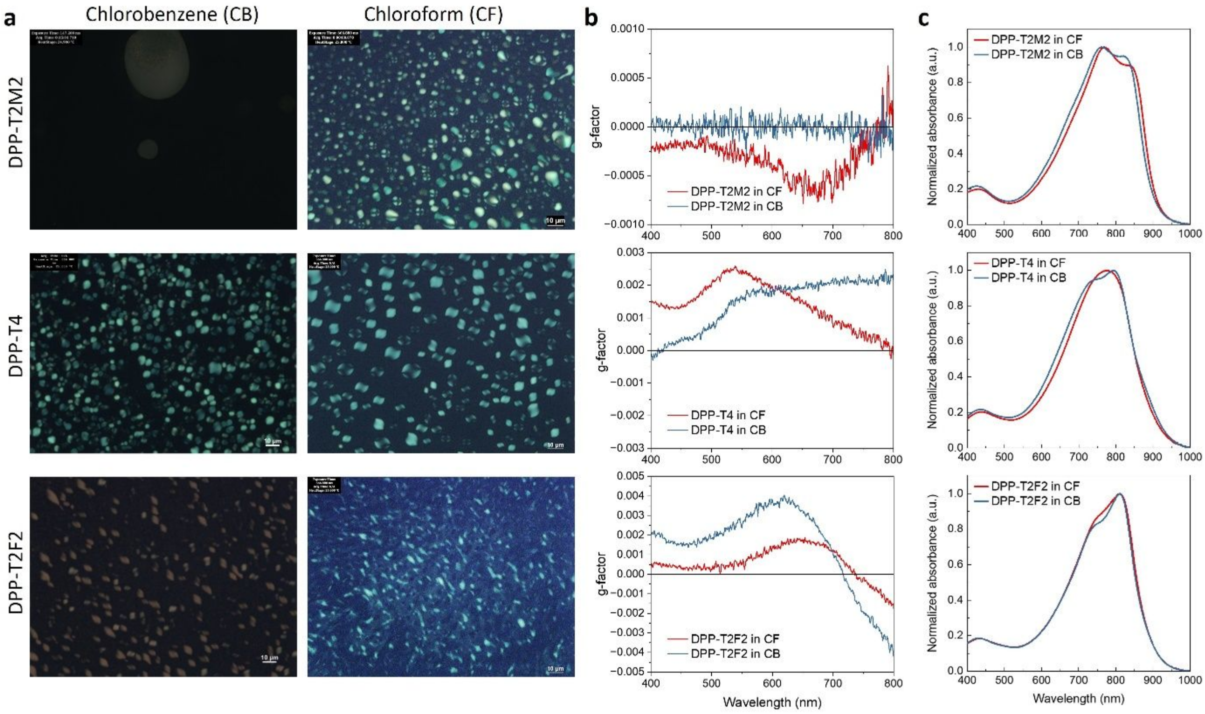

Figure S6. (a) Cross-polarized optical microscope images of 60 mg/ml DPP-T2M2 (top), DPP-T4 (middle) and DPP-T2F2 (bottom) prepared with CB and CF solvent. (b) CD spectra of the corresponding DPP solutions shown in a. (c) UV-VIS absorption spectra of 10 mg/ml DPP solutions.

We have tested how the solvent polarity can affect the solution aggregation and thus, the chiral helical assembly by comparing two solution systems with chlorobenzene (CB, polarity index: 2.7) and chloroform (CF, polarity index: 4.1). In the case of DPP-T4 and DPP-T2F2, both CB and CF solution systems clearly display birefringent microdroplets (tactoids), indicating a transition state where liquid crystalline mesophases nucleate and grow from an isotropic phase. In both solvents, these mesophases exhibit chirality as indicated by the CD signals. On the other hand, DPP-T2M2 produces crystalline mesophase only in CF system at the concentration of 60 mg/ml. We note that the DPP-T2M2 solution in CB prepared up to ~100 mg/ml displays no birefringence or apparent aggregates under the microscopy (Figure 2 in the main manuscript). The DPP-T2M2 solution concentration further increased around ~160 mg/ml begins to show a uniform birefringent feature. Figure S6 (b) shows the CD spectra of the corresponding DPP solutions shown in Figure S6 (a), confirming the chirality character matches with the mesophases observed under the microscopy. The question may be raised then what factor causes the drastic difference of DPP-T2M2 between in CF and CB system, and what is a general relationship between solution-state aggregation and chiral helical assembly.

While further in-depth studies should be needed to better understand this relationship, we have some hints from the simple experiment with UV-VIS absorption spectroscopy. Figure S6 (c) shows the UV-VIS absorption spectra of 10 mg/ml DPP pristine solutions prepared in CF and CB. DPP-T2M2 solution in CF is slightly more aggregated when compared to the CB system as its first vibronic peak around 850 nm is red-shifted, and further the (0-1)/(0-0) ratio is obviously higher. This suggests that the more aggregated DPP-T2M2 in CF may act as seeds, enabling the formation of chiral helical liquid crystals at substantially lower concentrations. In contrast, the spectra of both CF and CB systems for DPP-T4 and DPP-T2F2 are barely changed so their solution aggregation types may not affect the chiral assembly in both solvents.

Table S3. Handedness probability and g-factor comparison as a function of solution concentration.

**T2M2**	**60 mg/ml**	**100 mg/ml**	**160 mg/ml**	**200 mg/ml**
**Handedness probability**	-	60% LH	60% LH	66% RH
**g-factor**	-	0.0017 ± 0.0016	0.0045 ± 0.0035	0.010 ± 0.0071

**T4**	**60 mg/ml**	**100 mg/ml**	**160 mg/ml**	**200 mg/ml**
**Handedness probability**	53% LH	66% RH	60% LH	66% RH
**g-factor**	0.0013 ± 0.00080	0.0041 ± 0.0027	0.0082 ± 0.0062	0.0081 ± 0.0055

**T2F2**	**60 mg/ml**	**100 mg/ml**	**160 mg/ml**	**200 mg/ml**
**Handedness probability**	53% LH	53% LH	66% LH	73% RH
**g-factor**	0.0049 ± 0.0025	0.0097 ± 0.0031	0.038 ± 0.018	0.047 ± 0.035

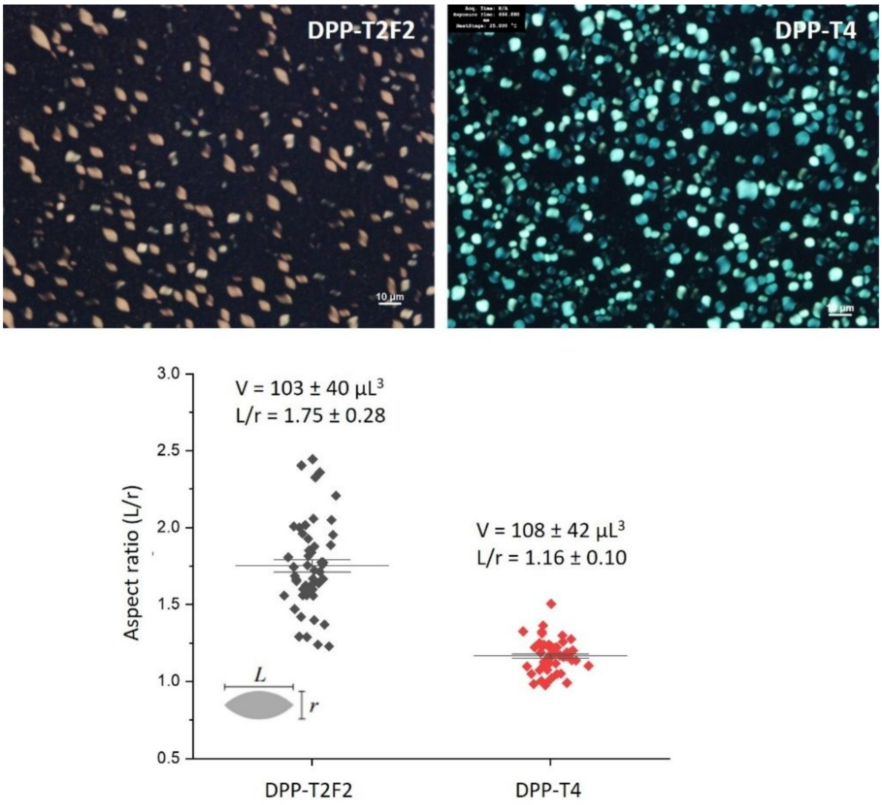

Figure S7. The aspect ratio comparison between DPP-T4 and DPP-T2F2 tactoids upon a similar solution volume of tactoids

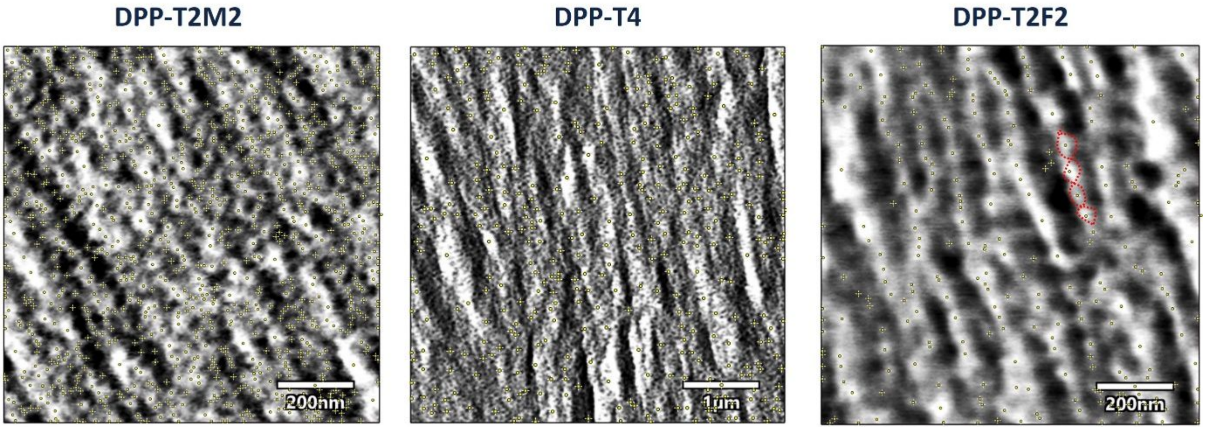

Figure S8. AFM phase images of freeze-dried DPP mesophase at 160 mg/ml. The red dotted lines in images exhibit twisted structures for DPP-T2F2 as an example. Estimating the helical pitch length of nanoscale fibers was performed by ImageJ. The small points on the phase images were found via a 'find maxima' function in ImageJ. The set value of prominence is > 10.00 for T2M2 and T2F2, and >70.00 for T4. Assuming these maxima points indicate the domains between the twisting points, the length between two adjacent points along the fiber longitudinal axis can be a half pitch length.

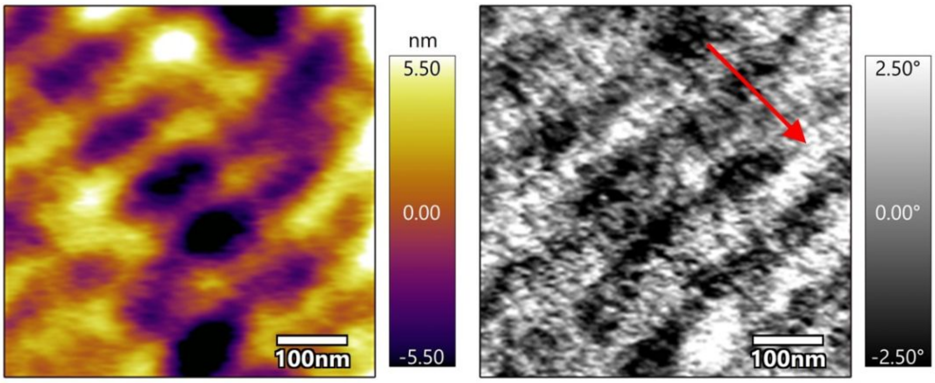

Figure S9. Zoom-in AFM topography (left) and phase (right) images of the DPP-T2M2 striped twist-bent phase shown in Figure 4d of the main text. The phase image clearly shows the fiber alignment direction, presumably polymer chain direction as well as indicated by red arrow.

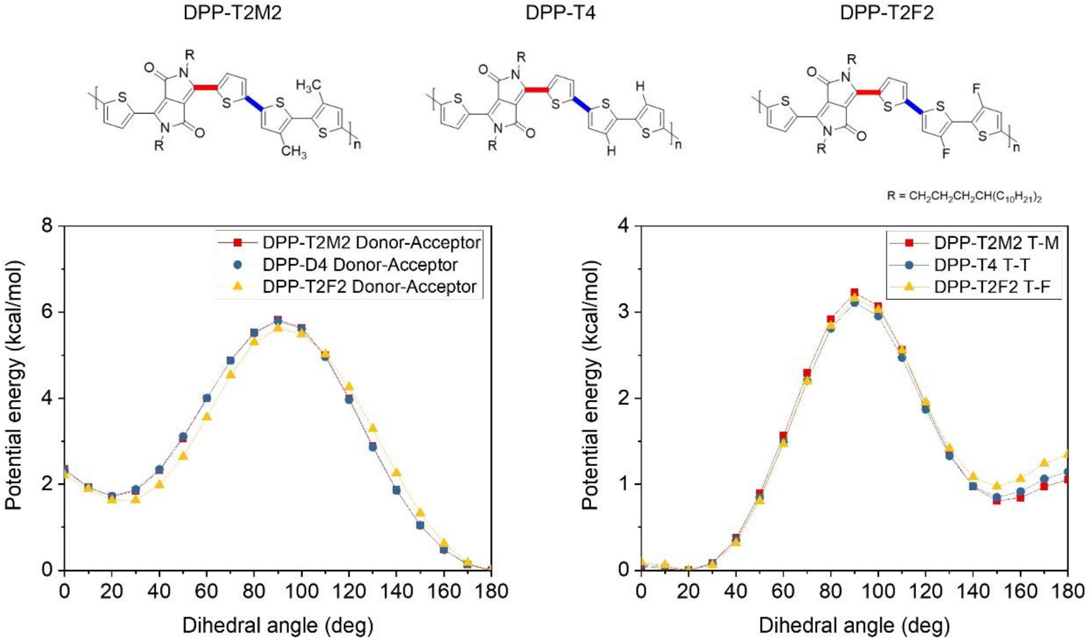

Figure S10. Potential energy plots of dihedral angles (left) between donor and acceptor moieties, indicated by the red line in the molecular structures, and (right) between two adjacent thiophenes close to the DPP unit, indicated by the blue line.

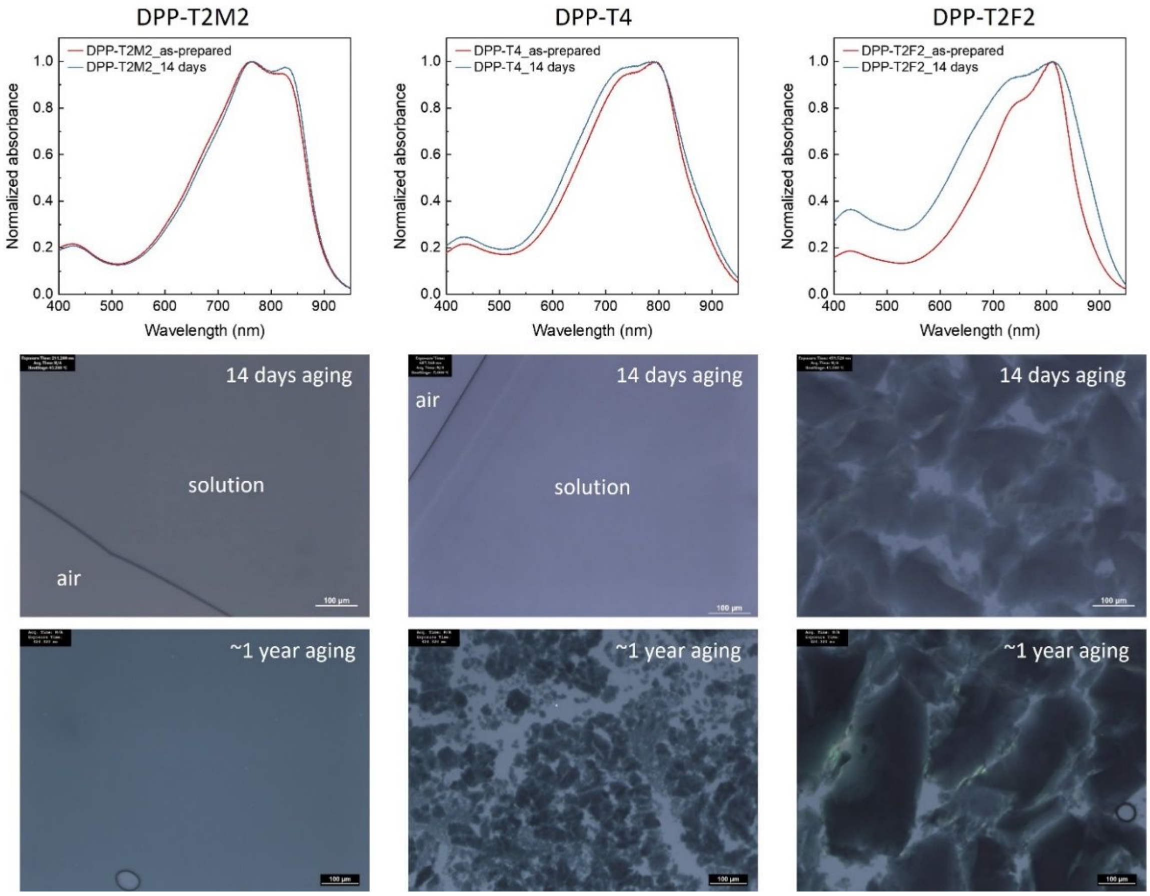

Figure S11. (Top) UV-VIS absorption spectra of as-prepared and 14-day aged 10 mg/ml DPP solutions. (Middle and Bottom) Cross-polarized optical microscope images of 10 mg/ml DPP solutions aged for 14 days and about 1 year, respectively.

Figure S11 shows an example of UV-VIS spectra and optical microscopy images of freshly prepared vs aged DPP solutions in chlorobenzene. The large broadening of UV-VIS spectra as well as apparent aggregation seen under the microscopy clearly indicate that DPP-T2F2 is highly aggregated, and the extent of aggregation depends on the solution aging time. When comparing DPP-T2M2 and DPP-T4, both were barely changed for 14 days, however DPP-T4 was also highly aggregated in the end after about one year. This observation is in line with our DFT calculations and hypotheses in the main text: more torsional DPP-T2M2 stack loosely to form more flexible 1D fibers and result in shorter helical pitch, whereas more planar DPP-T2F2 forms rigid 1D fibers with more ordered internal packing to yield longer helical pitch and long-range order. DPP-T4 likely sits in between DPP-T2M2 and DPP-T2F2. Accordingly, we observed that kinetics of solution aggregates goes as DPP-T2M2< DPP-T4 < DPP-T2F2, which is directly related to their propensity to form ordered solution state aggregates.

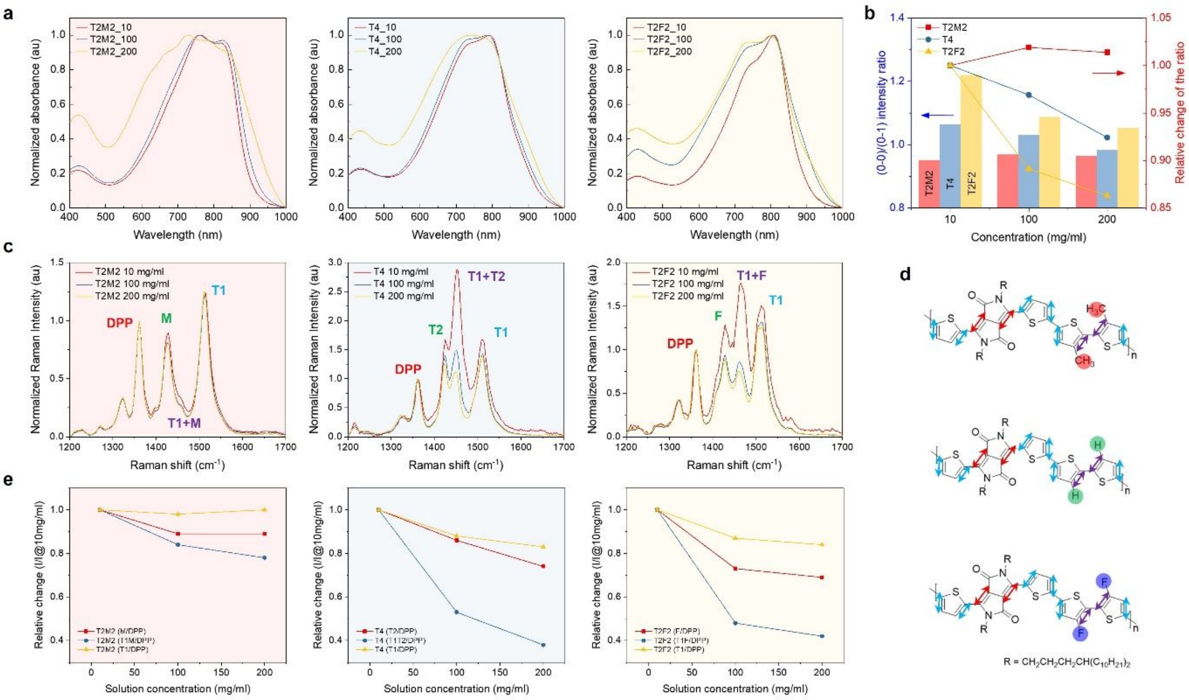

Figure S12. Molecular conformations of DPP mesophase by UV-VIS absorption and Raman spectroscopy. (a) Normalized UV-Vis absorption spectra of DPP solutions at 10, 100 and 200 mg/ml. (b) (0–0)/(0–1) peak ratio (bars) and the ratio change relative to the achiral, isotropic solution at 10 mg/ml (dot lines). The decrease of the ratio indicates the twisted molecular conformation as concentration increases. (c) Raman spectra of freeze-dried samples prepared at 10, 100 and 200 mg/ml. The peak intensity is normalized by the DPP peak around 1360 cm^{-1} by assuming its fused rings are relatively less changed compared to other bonds upon the concentration increases. (d) Illustration of representative Raman-active vibrational modes for each DPP polymer. The peak around 1360 cm^{-1} is assigned as strong localized C═C stretching in DPP units (red). The peak around 1520 cm^{-1} is assigned as delocalized C═C stretching over thiophene rings (cyan). The peaks around 1420 and 1460 cm^{-1} are assigned as localized C═C stretching in thiophene units with each different function. These functional peaks are the most drastic changes as concentration increases. (e) Raman spectra change relative to the achiral, isotropic solution at 10 mg/ml. The decrease of the intensity indicates twisted molecular conformation as concentration increases, in line with UV-VIS absorption results.

Supplementary Movie 1-3. In-situ cross polarized optical microscopy (CPOM) movies of 100 mg/ml DPP-T2M2 (Movie 1), DPP-T4 (Movie 2) and DPP-T2F2 (Movie 3) polymer solutions monitored during thermal heating and cooling. The sample was created by sandwiching the polymer solution between two thin glass slides. The initial solution for all DPP systems is birefringent resulting from blending/shearing the solution in the drop-and-dry process. Movie 1 shows that DPP-T2M2 birefringent feature disappears under heating and cooling, indicating the solution at 100 mg/ml is isotropic at the equilibrium state. In contrast, DPP-T4 (Movie 2) and DPP-T2F2 (Movie 3) result in the crystalline LC mesophase at their equilibrium state. However, their phase transition behavior during the thermal annealing and the shape of tactoids are notably distinct. With increasing the temperature, we observed a unique phenomenon to DPP-T4 where the aggregates are first wrinkling, then cracking, followed by segregating. In the case of DPP-T2F2, no such phenomenon was observed but instead the aggregation directly transitioned to LC phase when exceeding its critical temperature of around 85 °C. After all aggregates progress to the isotropic phase at 110 °C, we observed the nucleation and growth of DPP-T4 and DPP-T2F2 liquid crystals upon cooling to 25 °C.

References

(S1) Kwok, J. J.; Park, K. S.; Patel, B. B.; Dilmurat, R.; Beljonne, D.; Zuo, X.; Lee, B.; Diao, Y. Understanding Solution State Conformation and Aggregate Structure of Conjugated Polymers via Small Angle X-Ray Scattering. *Macromolecules* **2022**, *55* (11), 4353–4366.

(S2) Park, K. S.; Xue, Z.; Patel, B. B.; An, H.; Kwok, J. J.; Kafle, P.; Chen, Q.; Shukla, D.; Diao, Y. Chiral Emergence in Multistep Hierarchical Assembly of Achiral Conjugated Polymers. *Nat Commun* **2022**, *13* (1), 2738.

(S3) Albano, G.; Pescitelli, G.; Di Bari, L. Chiroptical Properties in Thin Films of π-Conjugated Systems. *Chem. Rev.* **2020**, *120* (18), 10145–10243.

(S4) Hirschmann, M.; Merten, C.; Thiele, C. M. Treating Anisotropic Artefacts in Circular Dichroism Spectroscopy Enables Investigation of Lyotropic Liquid Crystalline Polyaspartate Solutions. *Soft Matter* **2021**, *17* (10), 2849–2856.

(S5) Tivol, W. F.; Briegel, A.; Jensen, G. J. An Improved Cryogen for Plunge Freezing. *Microsc Microanal* **2008**, *14* (5), 375–379.
